# Temporal Trends of Asthma Among Children in the Western Pacific Region From 1990 to 2045: Longitudinal Observational Study

**DOI:** 10.2196/55327

**Published:** 2024-03-14

**Authors:** Cheng-hao Yang, Xin-yu Li, Jia-jie Lv, Meng-jie Hou, Ru-hong Zhang, Hong Guo, Chu Feng

**Affiliations:** 1 Department of General Surgery, School of Medicine Shanghai Putuo People's Hospital Tongji University Shanghai China; 2 Department of Plastic and Reconstructive Surgery Shanghai Ninth People's Hospital Shanghai Jiao Tong University School of Medicine Shanghai China; 3 Department of Gynecology and Obstetrics Tongji Hospital Tongji University School of Medicine Shanghai China

**Keywords:** allergic disorders, global burden of disease, disability-adjusted life years, DALYs, incidence, prevalence

## Abstract

**Background:**

Asthma has become one of the most common chronic conditions worldwide, especially among children. Recent findings show that the prevalence of childhood asthma has increased by 12.6% over the past 30 years, with >262 million people currently affected globally. The reasons for the growing asthma epidemic remain complex and multifactorial.

**Objective:**

This study aims to provide an up-to-date analysis of the changing global and regional asthma prevalence, mortality, disability, and risk factors among children aged <20 years by leveraging the latest data from the Global Burden of Disease Study 2019. Findings from this study can help inform priority areas for intervention to alleviate the rising burden of childhood asthma globally.

**Methods:**

The study used data from the Global Burden of Disease Study 2019, concentrating on children aged 0 to 14 years with asthma. We conducted an in-depth analysis of asthma, including its age-standardized prevalence, incidence, mortality, and disability-adjusted life years (DALYs), across diverse demographics, such as region, age, sex, and sociodemographic index, spanning 1990 to 2019. We also projected the future burden of the disease.

**Results:**

Overall, in the Western Pacific Region, the age-standardized prevalence rate of asthma among children increased slightly, from 3898.4 cases per 100,000 people in 1990 to 3924 per 100,000 in 2019. The age-standardized incidence rate of asthma also increased slightly, from 979.2 to 994.9 per 100,000. In contrast, the age-standardized death rate of asthma decreased from 0.9 to 0.4 per 100,000 and the age-standardized DALY rate decreased from 234.9 to 189.7 per 100,000. At the country level, Japan experienced a considerable decrease in the age-standardized prevalence rate of asthma among children, from 6669.1 per 100,000 in 1990 to 5071.5 per 100,000 in 2019. Regarding DALYs, Japan exhibited a notable reduction, from 300.6 to 207.6 per 100,000. Malaysia also experienced a DALY rate reduction, from 188.4 to 163.3 per 100,000 between 1990 and 2019. We project that the burden of disease in countries other than Japan and the Philippines will remain relatively stable up to 2045.

**Conclusions:**

The study indicates an increase in the prevalence and incidence of pediatric asthma, coupled with a decrease in mortality and DALYs in the Western Pacific Region between 1990 and 2019. These intricate phenomena appear to result from a combination of lifestyle shifts, environmental influences, and barriers to health care access. The findings highlight that nations such as Japan have achieved notable success in managing asthma. Overall, the study identified areas of improvement in view of persistent disease burden, underscoring the need for comprehensive collaborative efforts to mitigate the impact of pediatric asthma throughout the region.

## Introduction

Asthma, which affects >14% of children globally, is one of the most common chronic respiratory diseases and poses significant public health challenges [1]. Childhood asthma often starts early in life and persists throughout life, frequently continuing into adulthood [2]. Asthma symptoms manifest as wheezing, chest tightness, and coughing that disrupt daily functioning [3]. This disease leads to impaired quality of life, loss of school days, poor sleep, and increased health care use and costs [4]. The global burden and impact of childhood asthma continues to increase. Recent findings from the Global Burden of Disease (GBD) Study 2019 show that over the past 30 years, the prevalence of childhood asthma has increased by 12.6%, with >262 million people currently affected worldwide [5]. The reasons for the growing asthma epidemic remain complex and multifactorial and are linked to rising air pollution, urbanization, tobacco smoke exposure, genetics, overuse of antibiotics, and other modern lifestyle factors [6,7]. Careful analysis of recent global and regional trends in childhood asthma prevalence, morbidity, mortality, and projections can inform priority areas for intervention to alleviate this rising disease burden.

The Western Pacific Region (WPR), 1 of the 6 regions classified by the World Health Organization, comprises approximately one-fourth of the world’s population and faces a substantial pediatric disease burden owing to respiratory conditions. Previous analyses of global data reflect increasing asthma prevalence and mortality rates in the WPR from 1990 to 2015, with high rates among boys [8,9]. Recent WPR data on the burden of chronic respiratory diseases also suggest an increasing prevalence of asthma, although mortality rates may be declining [10,11]. Recent global data on pediatric asthma are limited, with most large-scale studies only presenting analyses up to 2015. Adding the latest available disease estimates from 2016 to 2019 provides contemporary 30-year perspective that is critical to uncovering current trends. Moreover, forecasting future patterns up to 2045 based on recent data can help anticipate trajectory changes and inform priority setting for pediatric asthma care in the WPR over the next 2 decades.

In this study, we leveraged the extensive global disease data within the GBD Study 2019 to analyze past trends from 1990 to 2019 and project estimates up to 2045 for key childhood asthma outcomes in the WPR. The findings can highlight success and gaps in managing this disease, whereas the future projections may guide strategic priorities for improving diagnosis, treatment access, and risk factor reduction to curb the rising prevalence of pediatric asthma across the region.

## Methods

### Data Source and Definitions

Our study used data from the GBD Study 2019 [12]. This extensive data set encompasses information about 369 diseases and injuries and 87 risk factors across 204 countries and territories, as described in a publication [13]. In the context of the GBD Study 2019, asthma was categorized based on the International Classification of Disease codes. Consistent with the most recent criteria set by the World Bank, countries within the WPR were categorized into 3 economic tiers: high income, upper middle income, and lower middle income. In each economic category, we selected 2 countries for our analysis, intentionally omitting islands or relatively isolated nations owing to their unique health care systems and health technology factors that might skew asthma burden data. For the high-income category, Japan and Singapore were chosen. China and Malaysia represent the upper middle-income tier, and Vietnam and the Philippines were the choices for the lower middle-income group.

We focused our analysis on the impact of asthma among children by dividing the patient data into 3 distinct age groups: <5 years, between 5 and 9 years, and between 10 and 14 years. We used linear regression methods to calculate the estimated annual percentage change (EAPC), providing an in-depth view of the changing trends. EAPC is commonly used in epidemiological studies to examine time-related changes in age-standardized rates (ASRs) of diseases. In this model, y represents ln (ASR) and x represents the calendar years. The EAPC, along with its 95% CI, was ascertained through the subsequent linear regression model [14]:

y= α + βx + ε

EAPC= 100 × (exp[β]−1)

To understand the ASR trend, we examined the EAPC and its 95% CI. An upward trend in the ASR is inferred when both the EAPC and the lower bound of the 95% CI are positive. In contrast, a downward trend is indicated when both the EAPC and the upper bound of the 95% CI are negative [14].

### Age-Period Cohort Analysis

In our study, we implemented the age-period cohort (APC) model, a highly esteemed statistical framework prevalent in health and social science disciplines. This model was instrumental in our analysis of disease prevalence across various time frames, with a particular emphasis on age, period, and birth cohort [15]. The APC model’s widespread use is especially notable in the study of noncommunicable diseases within the field of epidemiology. In this context, the age effect denotes the varying risk levels across different age groups. The period effect reflects the variations in disease incidence over time, affecting all age groups. Finally, the cohort effect pertains to the shifts in ratios among groups born in the same year, thereby affecting successive age groups over different periods. This nuanced approach enables a comprehensive understanding of disease dynamics across demographic, sociological, and epidemiological perspectives. Our approach involved using asthma data and applying the APC model to understand patterns and trends. To ensure precise analysis, we aligned 5-year age intervals with 5-year periods, spanning from 1990 to 2019, divided into 6 segments: 1990 to 1994, 1995 to 1999, 2000 to 2004, 2005 to 2009, 2010 to 2014, and 2015 to 2019. In addition, we defined 8 partially overlapping 10-year birth cohorts: 1975 to 1984, 1980 to 1989, 1985 to 1994, 1990 to 1999, 1995 to 2004, 2000 to 2009, 2005 to 2014, and 2010 to 2019. This arrangement facilitated a comprehensive exploration of how age, period, and cohort factors influence trends in congenital birth defects.

The APC model provides a refined method for examining disease burden, revealing both broad temporal trends and age-specific nuances in the disease data. The model identifies the general temporal trend or “net drift” as the annual percentage change in the disease burden. This reflects the combined impact of time progression and successive birth cohorts. In contrast, the “local drift” or age-specific temporal trend is the annual percentage change within each age group.

Even a small drift value, when expressed as a yearly percentage change, can indicate significant changes in disease prevalence over a 30-year period. We used a Wald chi-square test to evaluate the statistical significance of these trends. In the APC framework, age effects are observed through age-specific rates over different birth cohorts, adjusted for period influences. Period and cohort effects are shown as relative risks of the disease burden, comparing age-specific rates across different periods or cohorts with a selected reference point. This reference choice is arbitrary and does not alter the interpretability of the findings.

### Measurement of Health Inequalities

Total disability-adjusted life years (DALYs) and their ASRs were collected for assessing inequality. The analysis was conducted using the slope index of inequality and the concentration index. These are established measures for assessing absolute and relative gradient inequalities, respectively [16]. The slope index of inequality was calculated by performing a regression analysis on country-level, age-standardized, years of life lost rates owing to asthma among children against an income-based, social position scale. This scale was defined using the midpoint of cumulative population intervals, ranked by per capita gross domestic product. To address variations in the data and the diminishing marginal utility reflected in the data, we used a weighted regression model and applied logarithmic transformation to the relative social position values.

The health inequality concentration index was determined by plotting a Lorenz concentration curve. This curve represented the cumulative relative distribution of the population, ordered by income, against the years of life lost owing to asthma. We then quantified the inequality by numerically integrating the area beneath this curve. When the Lorenz curve is positioned above the line of equality, it indicates that the health burden predominantly affects low-income countries, a scenario reflected by a negative concentration index. It is proposed that an absolute value within the range of 0.2 to 0.3 signifies a considerably high degree of relative inequality.

This approach provided a nuanced understanding of the disparity in asthma burden among children across different income levels in the WPR [17]. The APC analysis was performed with the *apc_ie* package in Stata (version 17.0; StataCorp).

### Future Burden

To forecast the burden from 1990 to 2045, we used a log-linear APC model, which is adept at tempering exponential growth and confining linear trend extrapolation [18]. This makes it particularly effective for aligning with recent trends. The implementation of this model was conducted using the *Nordpred* package in R (R Foundation for Statistical Computing) [19], which has demonstrated effective empirical performance in forecasting future trends [20]. This method facilitates a more controlled and realistic projection of future trends, using past and present data.

### Ethical Considerations

The original GBD study obtained informed consent from the study participants or was granted exemptions by the institutional review board of the University of Washington. As this was a secondary analysis of existing data, no additional human participant research ethics review or informed consent was required. The data used in our study were thoroughly anonymized and deidentified to safeguard participant privacy and confidentiality. Furthermore, our analysis adheres to the established Guidelines on Accurate and Transparent Health Estimate Reporting [21-23]. All participants in this study consented to publication of the results.

## Results

### Temporal Trends in Pediatric Asthma Prevalence, Incidence, Deaths, and DALYs in the WPR

**Figure 1:**
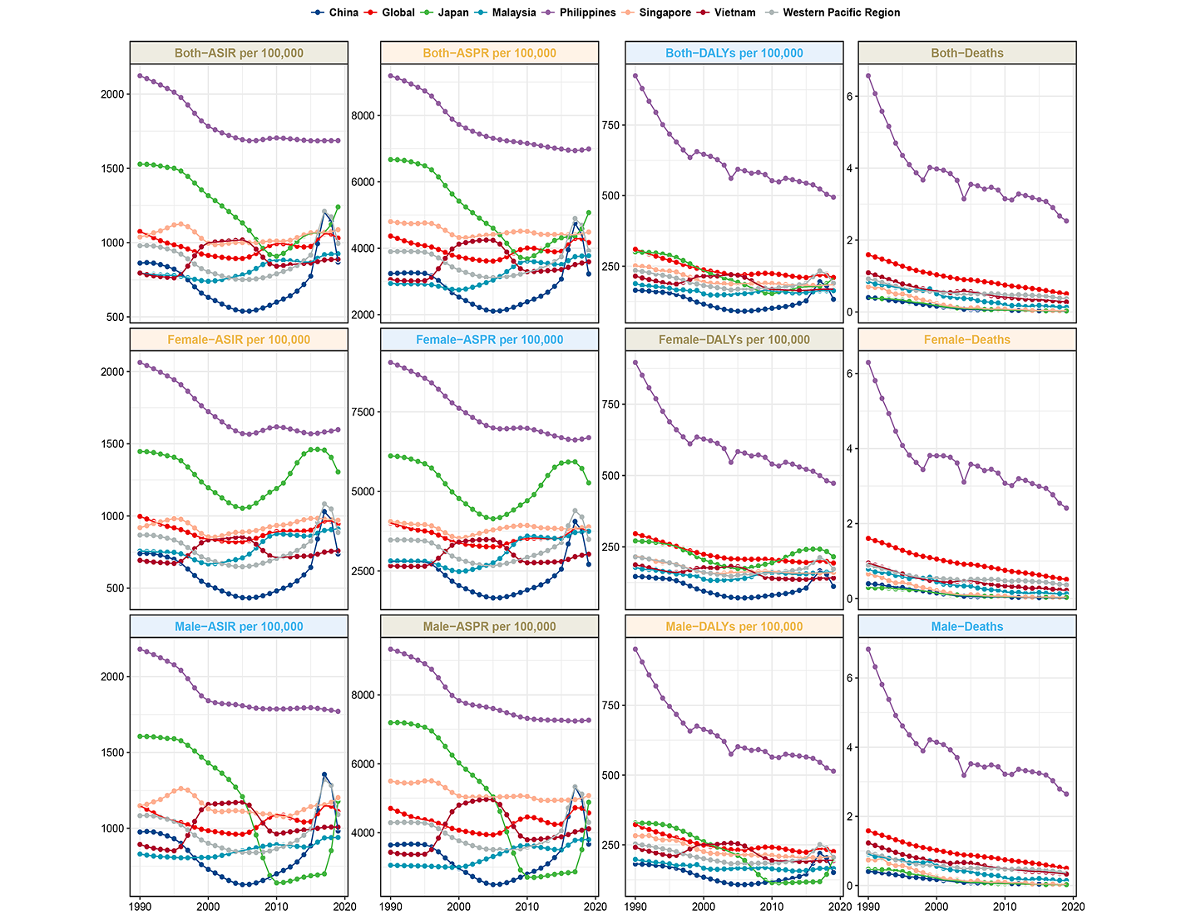
Temporal trends in pediatric asthma prevalence, incidence, deaths, and disability-adjusted life years (DALYs) in the Western Pacific Region (1990-2019). ASIR: age-standardized incidence rate; ASPR: age-standardized prevalence rate.

Overall, in the WPR, the age-standardized prevalence rate (ASPR) of asthma among children increased slightly from 3898.4 cases per 100,000 people in 1990 to 3924 per 100,000 in 2019. The age-standardized incidence rate (ASIR) of asthma also increased slightly from 979.2 to 994.9 per 100,000. In contrast, the age-standardized death rate (ASDR) of asthma decreased from 0.9 to 0.4 per 100,000, and the age-standardized DALYs rate decreased from 234.9 to 189.7 per 100,000 (Table 1; Figure 1).

At the country level, Australia had the highest ASPR of asthma in 1990 at 14,769.1 per 100,000, but it decreased to 10,974.9 per 100,000 in 2019 (EAPC=−1.49, 95% CI −1.67 to −1.31), whereas Mongolia and the Lao People’s Democratic Republic had the lowest ASPR at 2494.4 and 2604.4 per 100,000, respectively. Japan experienced a considerable decrease in the ASPR of asthma among children from 6669.1 per 100,000 in 1990 to 5071.5 per 100,000 in 2019 (EAPC=−1.91, 95% CI −2.36 to −1.45).

**Table 1:** Temporal trends in pediatric asthma prevalence, incidence, deaths, and disability-adjusted life years (DALYs) in the Western Pacific Region (1990-2019).

Category and location			Num_1990^a^ (95% UI^b^)		ASR_1990^c^ (95% UI)		Num_2019^d^ (95% UI)		ASR_2019^e^ (95% UI)		EAPC^f^ (95% CI)
**Prevalence**											
	Global	76,571,140.6 (55,413,704.2 to 109,195,913.6)		4365.6 (3159.3 to 6225.7)		81,720,617.8 (58,030,923.8 to 117,037,165.1)		4170 (2961.2 to 5972.1)		−0.04 (−0.28 to 0.2)	
	Western Pacific Region	16,959,863.9 (11,743,336 to 24,831,978.1)		3898.4 (2699.4 to 5707.9)		13,071,953.9 (8,866,483.2 to 19,389,884.5)		3924 (2661.6 to 5820.5)		0.13 (−0.38 to 0.64)	
	Australia	559,310.5 (481,247.9 to 632,619.3)		14,769.1 (12,707.7 to 16,704.8)		503,878 (360,666.4 to 698,506.1)		10,974.9 (7855.7 to 15,214.1)		−1.49 (−1.67 to −1.31)	
	Brunei Darussalam	3975.9 (2738.3 to 5794.5)		4384.9 (3020 to 6390.7)		4259.7 (2943.5 to 6287.9)		4478 (3094.4 to 6610.2)		−0.04 (−0.08 to 0.01)	
	Cambodia	132,886.6 (94,406.4 to 184,807.8)		2786 (1979.3 to 3874.6)		167,371.5 (115,385.3 to 247,138.7)		3325.3 (2292.4 to 4910.1)		0.59 (0.4 to 0.79)	
	China	10,454,057.7 (6,763,843.2 to 16,155,345.4)		3237.5 (2094.7 to 5003.1)		7,252,060.8 (4,725,473.7 to 11,276,601.5)		3226.3 (2102.3 to 5016.8)		0.18 (−0.73 to 1.09)	
	Cook Islands	340.7 (229.8 to 507.2)		5077.6 (3425.2 to 7558.7)		211.4 (143.2 to 316.2)		5053.9 (3423.3 to 7561.2)		0.21 (0.06 to 0.36)	
	Fiji	10,433.9 (7473.2 to 14,356)		3698.9 (2649.3 to 5089.3)		8548.6 (6045.2 to 12,147.8)		3219.8 (2276.9 to 4575.4)		−0.82 (−1.01 to −0.63)	
	Japan	1,538,373 (1,042,345.1 to 2,237,818.7)		6669.1 (4518.8 to 9701.3)		791,521.5 (519,208.1 to 1,202,911.3)		5071.5 (3326.7 to 7707.5)		−1.91 (−2.36 to −1.45)	
	Kiribati	1436 (1138.5 to 1834)		4919 (3900 to 6282.6)		1454.3 (1125 to 1901.6)		3471.6 (2685.5 to 4539.3)		−1.46 (−1.57 to −1.36)	
	Lao People’s Democratic Republic	59,551.2 (44,600.5 to 81,501)		3263.1 (2443.9 to 4465.9)		58,595.3 (42,118 to 81,854)		2604.4 (1872 to 3638.2)		−1.04 (−1.15 to −0.92)	
	Malaysia	193,359.9 (140,444.7 to 277,139.9)		2939.6 (2135.1 to 4213.2)		290,422.7 (195,058 to 437,283.4)		3774.5 (2535.1 to 5683.1)		1.12 (0.89 to 1.35)	
	Marshall Islands	804.7 (574.6 to 1122.8)		3628.4 (2591.1 to 5063)		671.8 (460.3 to 943.3)		3641.8 (2495.2 to 5113.6)		−0.07 (−0.4 to 0.25)	
	Micronesia	1668.2 (1208.3 to 2302.7)		3545.9 (2568.3 to 4894.6)		1231.8 (846.6 to 1773.8)		3921.6 (2695.3 to 5647.2)		0.31 (0.06 to 0.57)	
	Mongolia	20,810.5 (13,946.9 to 30,760.5)		2317.5 (1553.1 to 3425.5)		25,252.5 (16,220.4 to 38,449.7)		2494.4 (1602.2 to 3798)		0.13 (0.02 to 0.24)	
	Nauru	173.2 (119.6 to 246.5)		3897.4 (2692.3 to 5548.8)		165.2 (112.7 to 246.4)		4217.9 (2878.8 to 6292.8)		0.14 (−0.29 to 0.57)	
	New Zealand	90,992.3 (61,774.7 to 131,546.3)		11,373.9 (7721.7 to 16,443.1)		82,094.7 (55,753.8 to 118,776.2)		9200.2 (6248.2 to 13,311.1)		−0.91 (−1.06 to −0.76)	
	Niue	38.3 (26.6 to 55.6)		4622.7 (3204.8 to 6704.2)		19.2 (13.3 to 27.9)		4755.2 (3304 to 6935.6)		0.42 (0.18 to 0.65)	
	Palau	220.1 (153.7 to 317.5)		4643.7 (3242.7 to 6697)		153.1 (105.7 to 221.9)		4544.8 (3138 to 6586)		−0.09 (−0.25 to 0.07)	
	Papua New Guinea	90,389.7 (68,468.4 to 117,497.3)		5385.8 (4079.6 to 7000.9)		161,986.8 (123,526.9 to 209,990.4)		4401.1 (3356.1 to 5705.3)		−0.89 (−1.05 to −0.72)	
	Philippines	2,337,086.3 (1,695,954.1 to 3,260,644.5)		9192.4 (6670.7 to 12,825.1)		2,485,339.2 (1,817,569.4 to 3,472,416.2)		6987.9 (5110.4 to 9763.2)		−1.03 (−1.17 to −0.9)	
	Republic of Korea	456,884.3 (336,467.5 to 624,604.2)		3995.3 (2942.3 to 5462)		298,549.8 (200,075.2 to 448,074.1)		4353.2 (2917.3 to 6533.5)		0.8 (0.61 to 0.99)	
	Samoa	2419.2 (1684.8 to 3508.9)		3672.6 (2557.7 to 5326.9)		2777.5 (1915.5 to 4117)		3770.3 (2600.2 to 5588.6)		0.1 (−0.11 to 0.31)	
	Singapore	31,180.5 (23,196.2 to 42,797.1)		4801.6 (3572 to 6590.4)		35,086.6 (22,982.6 to 51,478.3)		4486.8 (2939 to 6583)		−0.26 (−0.36 to −0.15)	
	Solomon Islands	5537.9 (3822 to 7886.2)		3523.5 (2431.8 to 5017.7)		10,113.8 (7048.9 to 14,748.4)		3915.3 (2728.8 to 5709.4)		0.4 (0.06 to 0.73)	
	Tonga	1983.5 (1354.7 to 2930.9)		4990.1 (3408.2 to 7373.6)		1709.7 (1132.1 to 2545.2)		4764.5 (3154.9 to 7092.7)		−0.26 (−0.47 to −0.05)	
	Tuvalu	118.4 (83.5 to 166.5)		3587.8 (2532 to 5047.3)		155.3 (105.7 to 233.3)		4539.4 (3090.3 to 6821.3)		0.69 (0.35 to 1.02)	
	Vanuatu	2310.1 (1618.8 to 3262.5)		3452.8 (2419.6 to 4876.4)		4238.6 (2771.2 to 6182)		3824.1 (2500.2 to 5577.4)		0.17 (−0.3 to 0.63)	
	Vietnam	800,414.4 (552,891.5 to 1,200,763.8)		3055.4 (2110.6 to 4583.7)		765,718.1 (512,976.9 to 1,147,161.2)		3591.3 (2405.9 to 5380.3)		0.31 (−0.19 to 0.8)	
**Incidence**											
	Global	18,857,697.1 (12,709,797.1 to 26,396,036.1)		1075.1 (724.6 to 1504.9)		20,191,786.5 (13,397,981.9 to 28,406,953.9)		1030.3 (683.7 to 1449.5)		0.04 (−0.19 to 0.28)	
	Western Pacific Region	4,259,983.4 (2,755,425.9 to 6,210,138.5)		979.2 (633.4 to 1427.5)		3,314,337.9 (2,123,433.1 to 4,804,520.4)		994.9 (637.4 to 1442.2)		0.13 (−0.43 to 0.69)	
	Australia	70,584 (51,654 to 90,221.6)		1863.8 (1364 to 2382.4)		68,562.3 (46,626.2 to 94,480.1)		1493.4 (1015.6 to 2057.9)		−1.41 (−1.68 to −1.14)	
	Brunei Darussalam	956.1 (636.6 to 1361.6)		1054.4 (702.1 to 1501.7)		991.9 (645.9 to 1417.3)		1042.8 (679 to 1489.9)		−0.22 (−0.31 to −0.14)	
	Cambodia	36,302.6 (24,912.1 to 50,083.9)		761.1 (522.3 to 1050)		42,971.3 (28,972.3 to 61,579.7)		853.7 (575.6 to 1223.4)		0.46 (0.24 to 0.69)	
	China	2,784,848.8 (1,724,280.8 to 4,146,601.5)		862.4 (534 to 1284.1)		1,953,731.6 (1,219,396.4 to 2,922,245.1)		869.2 (542.5 to 1300.1)		0.13 (−0.81 to 1.08)	
	Cook Islands	84.4 (56.6 to 119)		1258 (842.8 to 1773.7)		50.9 (33.3 to 72)		1218.1 (795.8 to 1721.7)		0.08 (−0.05 to 0.21)	
	Fiji	2779.6 (1951 to 3821.7)		985.4 (691.7 to 1354.8)		2288.2 (1563.2 to 3304.5)		861.9 (588.8 to 1244.6)		−0.72 (−0.85 to −0.58)	
	Japan	352,531.4 (221,092.9 to 505,171.5)		1528.3 (958.5 to 2190)		193,514 (120,094.8 to 282,444.1)		1239.9 (769.5 to 1809.7)		−1.65 (−2.06 to −1.23)	
	Kiribati	369.9 (271.8 to 484.5)		1267 (931.1 to 1659.7)		393 (285.9 to 531.9)		938 (682.4 to 1269.6)		−1.2 (−1.26 to −1.14)	
	Lao People’s Democratic Republic	15,343.9 (11,014.5 to 20,628.6)		840.8 (603.5 to 1130.3)		15,570.3 (10,872.6 to 21,645.6)		692 (483.3 to 962.1)		−0.91 (−1.05 to −0.78)	
	Malaysia	52,256.7 (37,072.8 to 71,530.1)		794.4 (563.6 to 1087.4)		71,186.2 (47,019.1 to 102,929)		925.2 (611.1 to 1337.7)		0.7 (0.52 to 0.87)	
	Marshall Islands	213.7 (148.4 to 289.9)		963.6 (669.1 to 1307.1)		172.1 (116.6 to 235.3)		933 (632.2 to 1275.6)		−0.12 (−0.38 to 0.13)	
	Micronesia	444.9 (306.9 to 601.8)		945.6 (652.4 to 1279.1)		305.2 (202.4 to 423.3)		971.6 (644.4 to 1347.7)		0 (−0.23 to 0.23)	
	Mongolia	5950.8 (3718.1 to 8790.1)		662.7 (414.1 to 978.9)		7326.6 (4530.3 to 10,802.7)		723.7 (447.5 to 1067.1)		0.42 (0.24 to 0.6)	
	Nauru	47.2 (31.8 to 66.6)		1062 (715.6 to 1498.4)		42.3 (27.9 to 60.5)		1079.7 (713.3 to 1545.2)		−0.05 (−0.4 to 0.3)	
	New Zealand	16,691.4 (10,938 to 23,215)		2086.4 (1367.2 to 2901.8)		15,931.6 (10,669.5 to 22,810.8)		1785.4 (1195.7 to 2556.4)		−0.76 (−0.92 to −0.61)	
	Niue	9.5 (6.4 to 13.5)		1150.2 (772.5 to 1628.6)		4.6 (3 to 6.4)		1144.3 (748.7 to 1585.5)		0.28 (0.09 to 0.47)	
	Palau	55.1 (36.1 to 77.1)		1162.9 (761.7 to 1626)		36.6 (24.1 to 51)		1085.4 (715.3 to 1514.9)		−0.25 (−0.36 to −0.14)	
	Papua New Guinea	22,420.8 (16,186.6 to 29,807.9)		1335.9 (964.5 to 1776.1)		41,888.7 (30,890.2 to 55,842.8)		1138.1 (839.3 to 1517.2)		−0.75 (−0.87 to −0.63)	
	Philippines	539,771.3 (381,497.1 to 756,103.8)		2123.1 (1500.5 to 2974)		599,984.4 (418,410.1 to 845,919.5)		1686.9 (1176.4 to 2378.4)		−0.83 (−0.99 to −0.66)	
	Republic of Korea	102,439.2 (71,056 to 141,887.1)		895.8 (621.4 to 1240.8)		69,002.6 (44,555.3 to 100,627.1)		1006.1 (649.7 to 1467.3)		0.68 (0.59 to 0.77)	
	Samoa	615.6 (417 to 861.7)		934.5 (633.1 to 1308.2)		682.4 (444.4 to 980.7)		926.3 (603.3 to 1331.2)		−0.05 (−0.25 to 0.14)	
	Singapore	6739.1 (4680.5 to 9182.1)		1037.8 (720.8 to 1414)		8501.5 (5557 to 12,249.4)		1087.2 (710.6 to 1566.4)		−0.07 (−0.24 to 0.09)	
	Solomon Islands	1483.4 (989.2 to 2097)		943.8 (629.4 to 1334.3)		2632.6 (1794.4 to 3780.3)		1019.1 (694.6 to 1463.4)		0.3 (0.04 to 0.56)	
	Tonga	488 (324.7 to 687.4)		1227.8 (816.9 to 1729.3)		424.8 (272.8 to 601.7)		1183.7 (760.3 to 1676.8)		−0.21 (−0.4 to −0.02)	
	Tuvalu	32.4 (22.3 to 44.9)		981.8 (676.2 to 1359.6)		37.7 (24.9 to 53.4)		1103.2 (727 to 1560.3)		0.26 (−0.05 to 0.56)	
	Vanuatu	629.3 (438.4 to 879.6)		940.6 (655.3 to 1314.7)		1089.1 (696.2 to 1543.1)		982.6 (628.1 to 1392.2)		−0.01 (−0.4−0.39)	
	Vietnam	208,363.9 (137,308.5 to 301,488.3)		795.4 (524.2 to 1150.9)		189,272 (123,981.6 to 270,239.5)		887.7 (581.5 to 1267.5)		0.31 (−0.08 to 0.7)	
**Deaths**											
	Global	27,949.7 (18,502.8 to 35,060.2)		1.6 (1.1 to 2)		9911.2 (7949.9 to 12,337)		0.5 (0.4 to 0.6)		−3.62 (−3.73 to −3.52)	
	Western Pacific Region	4013 (2666.9 to 5029.9)		0.9 (0.6 to 1.2)		1228 (975.7 to 1472.8)		0.4 (0.3 to 0.4)		−2.44 (−2.77 to −2.11)	
	Australia	15.8 (13.3 to 18.6)		0.4 (0.4 to 0.5)		6.8 (5.3 to 8.7)		0.1 (0.1 to 0.2)		−3.86 (−4.33 to −3.38)	
	Brunei Darussalam	0.4 (0.3 to 0.6)		0.5 (0.3 to 0.7)		0.1 (0.1 to 0.1)		0.1 (0.1 to 0.2)		−5.46 (−5.9 to −5.01)	
	Cambodia	165.1 (68.1 to 248.5)		3.5 (1.4 to 5.2)		33.6 (22.8 to 51.5)		0.7 (0.5 to 1)		−5.4 (−5.54 to −5.26)	
	China	1299 (775.3 to 1681.3)		0.4 (0.2 to 0.5)		52.3 (39.8 to 73.1)		0 (0 to 0)		−9.95 (−10.44 to −9.45)	
	Cook Islands	0 (0 to 0)		0.5 (0.3 to 0.7)		0 (0 to 0)		0 (0 to 0.1)		−9.27 (−9.87 to −8.66)	
	Fiji	6.8 (4.9 to 9.6)		2.4 (1.7 to 3.4)		3.9 (2.7 to 5.3)		1.5 (1 to 2)		−2.68 (−3.28 to −2.08)	
	Japan	88.8 (70.7 to 98.7)		0.4 (0.3 to 0.4)		4.7 (3.3 to 5.9)		0 (0 to 0)		−9.85 (−10.31 to −9.39)	
	Kiribati	1.6 (1 to 2.3)		5.6 (3.5 to 8)		0.8 (0.5 to 1.4)		2 (1.3 to 3.3)		−3.52 (−3.73 to −3.31)	
	Lao People’s Democratic Republic	248.2 (98.6 to 403.9)		13.6 (5.4 to 22.1)		45.3 (27.7 to 68.7)		2 (1.2 to 3.1)		−6.35 (−6.49 to −6.21)	
	Malaysia	55.9 (37.2 to 77.7)		0.8 (0.6 to 1.2)		10.3 (6.8 to 16.1)		0.1 (0.1 to 0.2)		−6.73 (−7.13 to −6.33)	
	Marshall Islands	0.3 (0.2 to 0.5)		1.3 (0.8 to 2.1)		0.1 (0.1 to 0.2)		0.7 (0.5 to 1.1)		−2.4 (−3.07 to −1.72)	
	Micronesia	1 (0.6 to 1.4)		2 (1.4 to 3)		0.2 (0 to 0.3)		0.5 (0.1 to 0.8)		−5.14 (−5.3 to −4.97)	
	Mongolia	5 (1.1 to 12.4)		0.6 (0.1 to 1.4)		0.6 (0.3 to 1.1)		0.1 (0 to 0.1)		−8.01 (−8.48 to −7.54)	
	Nauru	0.1 (0.1 to 0.2)		2.5 (1.2 to 4.1)		0 (0 to 0.1)		0.9 (0.5 to 1.4)		−3.45 (−4.6 to −2.28)	
	New Zealand	4.3 (3.7 to 4.9)		0.5 (0.5 to 0.6)		1.6 (1.3 to 2)		0.2 (0.1 to 0.2)		−4.23 (−4.53 to −3.93)	
	Niue	0 (0 to 0)		1 (0.6 to 1.5)		0 (0 to 0)		0.5 (0.2 to 0.8)		−2.98 (−3.45 to −2.52)	
	Palau	0 (0 to 0.1)		0.7 (0.3 to 1.4)		0 (0 to 0)		0.2 (0.1 to 0.3)		−3.69 (−4.09 to −3.3)	
	Papua New Guinea	86.6 (33.8 to 152)		5.2 (2 to 9.1)		102.4 (48.8 to 181.5)		2.8 (1.3 to 4.9)		−1.84 (−2.1 to −1.57)	
	Philippines	1670.4 (1021.5 to 2195.8)		6.6 (4 to 8.6)		900.8 (652.1 to 1102.5)		2.5 (1.8 to 3.1)		−2.35 (−2.71 to −1.98)	
	Republic of Korea	59.6 (46.4 to 73.3)		0.5 (0.4 to 0.6)		1.2 (0.7 to 2.2)		0 (0 to 0)		−12.37 (−12.82 to −11.92)	
	Samoa	0.9 (0.6 to 1.4)		1.4 (0.9 to 2.1)		0.2 (0.1 to 0.4)		0.3 (0.2 to 0.5)		−4.6 (−4.8 to −4.4)	
	Singapore	4.5 (3.5 to 6)		0.7 (0.5 to 0.9)		0.3 (0.2 to 0.6)		0 (0 to 0.1)		−10.58 (−11.09 to −10.06)	
	Solomon Islands	2.1 (1.3 to 3.1)		1.3 (0.8 to 2)		1.6 (1.1 to 2.3)		0.6 (0.4 to 0.9)		−2.34 (−2.67 to −2.02)	
	Tonga	0.2 (0.2 to 0.3)		0.6 (0.4 to 0.9)		0.1 (0.1 to 0.1)		0.2 (0.2 to 0.4)		−3.17 (−3.65 to −2.69)	
	Tuvalu	0.1 (0.1 to 0.3)		4.5 (2.2 to 7.8)		0 (0 to 0)		0.4 (0.3 to 0.7)		−8 (−8.23 to −7.76)	
	Vanuatu	1.1 (0.7 to 1.8)		1.7 (1 to 2.7)		1.1 (0.7 to 1.7)		1 (0.6 to 1.6)		−1.96 (−2.34 to −1.57)	
	Vietnam	286.1 (153.9 to 446.1)		1.1 (0.6 to 1.7)		59.3 (37.2 to 88.2)		0.3 (0.2 to 0.4)		−4.25 (−4.47 to −4.02)	
**DALYs**											
	Global	5,433,991.4 (3,840,150.8 to 7,609,681.4)		309.8 (218.9 to 433.9)		4,116,909.5 (2,702,283.8 to 6,170,009.6)		210.1 (137.9 to 314.8)		−1.19 (−1.4 to −0.98)	
	Western Pacific Region	1,021,929.5 (690,976.1 to 1,505,850.2)		234.9 (158.8 to 346.1)		631,850.6 (400,999.9 to 980,983.6)		189.7 (120.4 to 294.5)		−0.46 (−0.92 to 0)	
	Australia	23,892.2 (15,923.8 to 34,403.6)		630.9 (420.5 to 908.5)		20,906.3 (12,111.7 to 33,982.2)		455.4 (263.8 to 740.2)		−1.58 (−1.77 to −1.39)	
	Brunei Darussalam	196.8 (125.4 to 303.9)		217.1 (138.3 to 335.2)		180.2 (105.2 to 299.2)		189.5 (110.6 to 314.5)		−0.55 (−0.63 to −0.47)	
	Cambodia	19,258.5 (10,582.7 to 27,408.2)		403.8 (221.9 to 574.6)		9547.6 (6473.9 to 14,223.4)		189.7 (128.6 to 282.6)		−2.6 (−2.83 to −2.37)	
	China	531,512.5 (333,439 to 832,409.3)		164.6 (103.3 to 257.8)		298,487.3 (166,283.3 to 506,739.3)		132.8 (74 to 225.4)		−0.57 (−1.53 to 0.39)	
	Cook Islands	16.3 (10.1 to 25.8)		243.4 (150.8 to 384.3)		8.7 (5 to 14.2)		207.2 (118.9 to 339.3)		−0.41 (−0.58 to −0.23)	
	Fiji	970.2 (732.1 to 1300.9)		344 (259.5 to 461.2)		660.6 (476 to 893.5)		248.8 (179.3 to 336.5)		−1.83 (−2.24 to −1.42)	
	Japan	69,344.6 (42,600 to 110,193.4)		300.6 (184.7 to 477.7)		32,395.6 (18,423.8 to 54,912.4)		207.6 (118 to 351.8)		−2.27 (−2.75 to 1.79)	
	Kiribati	193.6 (135.4 to 259.6)		663.2 (463.8 to 889.3)		127.7 (90.5 to 179)		304.8 (216.1 to 427.2)		−2.81 (−2.95 to −2.67)	
	Lao People’s Democratic Republic	23,364.8 (10,409.3 to 36,578.7)		1280.3 (570.4 to 2004.3)		6125.7 (4222.4 to 8678.5)		272.3 (187.7 to 385.7)		−5.29 (−5.38 to −5.21)	
	Malaysia	12,392.2 (8756.2 to 17,637.8)		188.4 (133.1 to 268.1)		12,563.3 (7558.1 to 20,841)		163.3 (98.2 to 270.9)		−0.37 (−0.6 to −0.15)	
	Marshall Islands	55.9 (39.4 to 81.7)		252.2 (177.6 to 368.2)		37.3 (25 to 54.7)		202.4 (135.5 to 296.7)		−1 (−1.22 to −0.77)	
	Micronesia	145.3 (105.1 to 198.1)		308.8 (223.3 to 421)		62 (39 to 95.4)		197.3 (124.1 to 303.7)		−1.8 (−2.09 to −1.52)	
	Mongolia	1254.3 (740.5 to 2033.9)		139.7 (82.5 to 226.5)		1068.3 (611.3 to 1777.2)		105.5 (60.4 to 175.5)		−1.27 (−1.5 to −1.04)	
	Nauru	16.1 (10.1 to 23.3)		362.9 (227.6 to 525.3)		9.6 (6.3 to 14.3)		244.9 (161.5 to 365.9)		−1.71 (−2.23 to −1.18)	
	New Zealand	4006.8 (2424.5 to 6422.7)		500.8 (303.1 to 802.8)		3441.8 (2050.7 to 5584.2)		385.7 (229.8 to 625.8)		−1.1 (−1.25 to −0.95)	
	Niue	2.2 (1.4 to 3.3)		266.1 (174.6 to 400.4)		0.9 (0.6 to 1.4)		229.6 (142.2 to 350.8)		−0.47 (−0.62 to −0.32)	
	Palau	11.8 (7.5 to 18)		248.3 (158.2 to 379.6)		6.7 (4 to 10.6)		199.2 (119.1 to 315.2)		−0.63 (−0.81 to −0.44)	
	Papua New Guinea	10,851.6 (6147.3 to 16,546.1)		646.6 (366.3 to 985.9)		15,023.5 (9532 to 21,930.5)		408.2 (259 to 595.8)		−1.48 (−1.68 to −1.28)	
	Philippines	234,992.5 (162,218.2 to 310,470)		924.3 (638.1 to 1221.2)		175,739.6 (129,991.3 to 238,440.4)		494.1 (365.5 to 670.4)		−1.71 (−1.95 to −1.48)	
	Republic of Korea	23,353.3 (15,712.7 to 34,903.5)		204.2 (137.4 to 305.2)		12,181.8 (6904.1 to 20,592.3)		177.6 (100.7 to 300.3)		−0.01 (−0.21 to 0.19)	
	Samoa	169.9 (117.4 to 241.1)		257.9 (178.3 to 366)		129.2 (80.3 to 208.1)		175.4 (109 to 282.5)		−1.22 (−1.48 to −0.96)	
	Singapore	1632.1 (1101.8 to 2404.2)		251.3 (169.7 to 370.2)		1444.6 (824.5 to 2402.1)		184.7 (105.4 to 307.2)		−1.1 (−1.32 to −0.88)	
	Solomon Islands	391.6 (269.3 to 554.7)		249.2 (171.3 to 352.9)		540.9 (365.9 to 809.9)		209.4 (141.6 to 313.5)		−0.63 (−0.85 to −0.41)	
	Tonga	99.8 (63.7 to 155.8)		251.1 (160.4 to 392)		76.3 (45.5 to 122.2)		212.6 (126.9 to 340.6)		−0.72 (−0.94 to −0.49)	
	Tuvalu	17.3 (10.3 to 27.2)		524.6 (311.8 to 825.4)		7.4 (4.6 to 11.7)		216.2 (134.3 to 340.8)		−3.17 (−3.71 to −2.63)	
	Vanuatu	184.8 (126 to 264.9)		276.2 (188.3 to 396)		257.7 (174.3 to 386.7)		232.5 (157.3 to 348.9)		−0.81 (−1.06 to −0.55)	
	Vietnam	56,279.1 (37,947.7 to 83,399.1)		214.8 (144.9 to 318.4)		35,963.9 (22,611.9 to 57,339.3)		168.7 (106.1 to 268.9)		−0.89 (−1.21 to −0.57)	

^a^Num_1990: number in 1990.

^b^UI: uncertainty interval.

^c^ASR_1990: age-standardized rate in 1990.

^d^Num_2019: number in 2019.

^e^ASR_2019: age-standardized rate in 2019.

^f^EAPC: estimated annual percentage change.

New Zealand had the highest ASIR of asthma in 1990 at 2086.4 per 100,000. By 2019, the incidence in New Zealand decreased to 1785.4 per 100,000 (EAPC=0.31, 95% CI −0.08 to 0.7). The countries with the lowest ASIR of asthma in 2019 were the Lao People’s Democratic Republic at 692 per 100,000 and Mongolia at 723.7 per 100,000.

Papua New Guinea had the highest ASDR of asthma in 2019 at 2.8 per 100,000. By 2019, the ASDR of asthma in the Lao People’s Democratic Republic decreased from 13.6 per 100,000 to 2 per 100,000 (EAPC=−6.35, 95% CI −6.49 to −6.21). In terms of DALYs owing to asthma, there was a significant overall decrease among children in the WPR. The figures decreased from 234.9 per 100,000 in 1990 to 189.7 per 100,000 in 2019, indicating an EAPC of −0.46, with the 95% CI ranging from −0.92 to 0. This trend mirrors the mortality patterns, with Japan exhibiting a notable reduction in pediatric asthma DALYs during this period. In Japan, the rate decreased from 300.6 to 207.6 per 100,000 (EAPC=−2.27, 95% CI −2.75 to −1.79). Malaysia also experienced a reduction, from 188.4 to 163.3 per 100,000, between 1990 and 2019 (EAPC=−0.37, 95% CI −0.60 to −0.15), as shown in Table 1 and Figure 1.

### Trends in Asthma Burden Among Children Across the Sociodemographic Index Quintiles in the WPR

**Figure 2:**
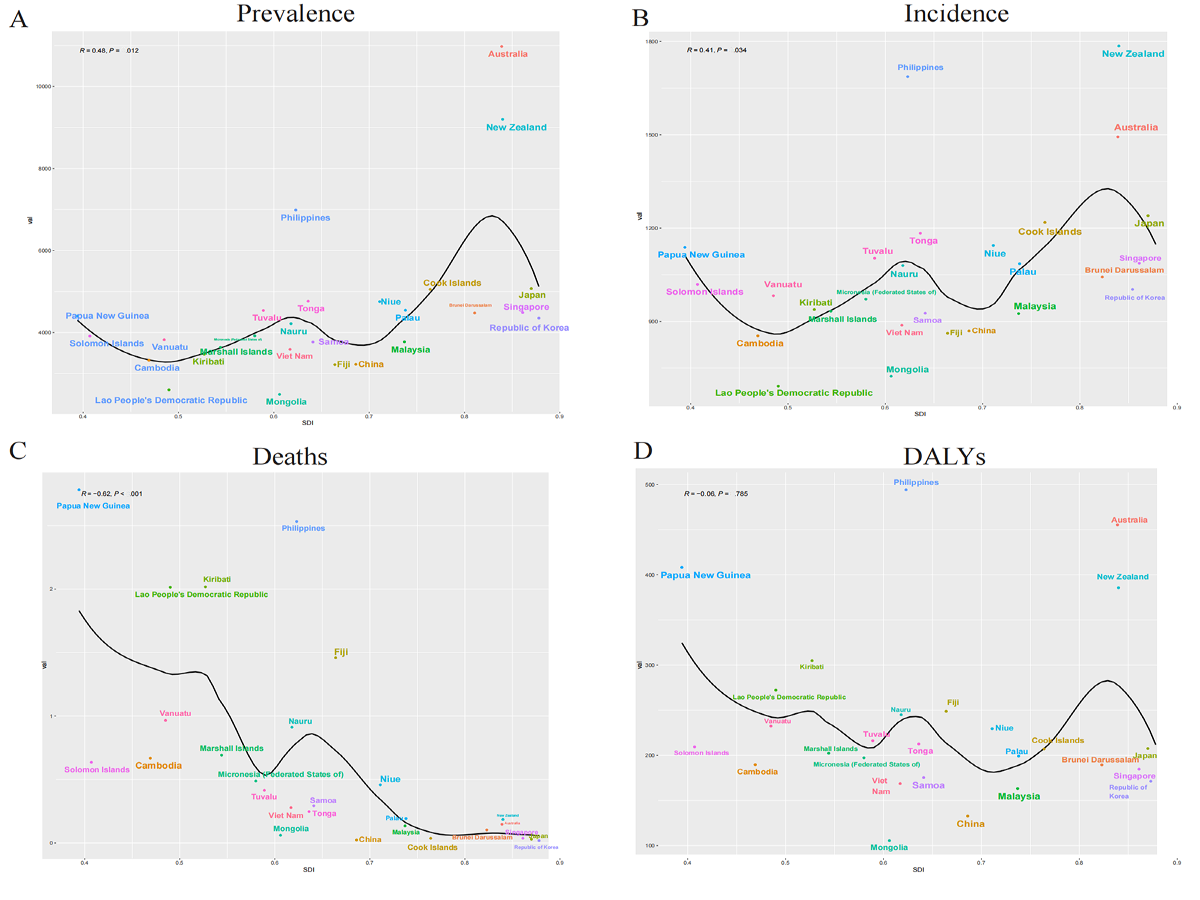
The relationship between sociodemographic index levels and the prevalence, incidence, deaths, and disability-adjusted life years (DALYs) of pediatric asthma (1990-2019). SDI: sociodemographic index.

Over the 3 decades under study, the WPR experienced significant changes in the ASPR, ASIR, and ASDR for asthma burden among children across all countries categorized by the sociodemographic index (SDI) level (Figure 2). We found that as the SDI increased, the ASPR and ASIR gradually increased, whereas the ASDR gradually decreased.

### Temporal Trends in Asthma Burden Among Children Across Age Groups

The annual percentage change in asthma prevalence for each age group, that is, the local trends in prevalence calculated using the APC model, is presented in Figure S1 in Multimedia Appendix 1. In the WPR, asthma prevalence decreased in the 0-to-4 year and 10-to-14 year age groups. The upper middle-income group (China and Malaysia) showed the same trend. Japan and Singapore also showed favorable changes, with Japan showing a gradual downward trend and Singapore maintaining a stable trend, with no significant upward trend. In Vietnam, asthma prevalence demonstrated decreasing trends in the 0-to-4 year and 5-to-9-year age groups and showed a flat trend in the 5-to-9-year and 10-to-14-year age groups. The local trends in prevalence are shown in Multimedia Appendix 2. Temporal changes in the age distribution of asthma prevalence are illustrated in Figure S2 in Multimedia Appendix 1. The distribution of age around the world and across the WPR has remained relatively stable over the past 30 years.

### Age, Period, and Birth Cohort Effects on Asthma Prevalence Across Different Age Groups

**Figure 3:**
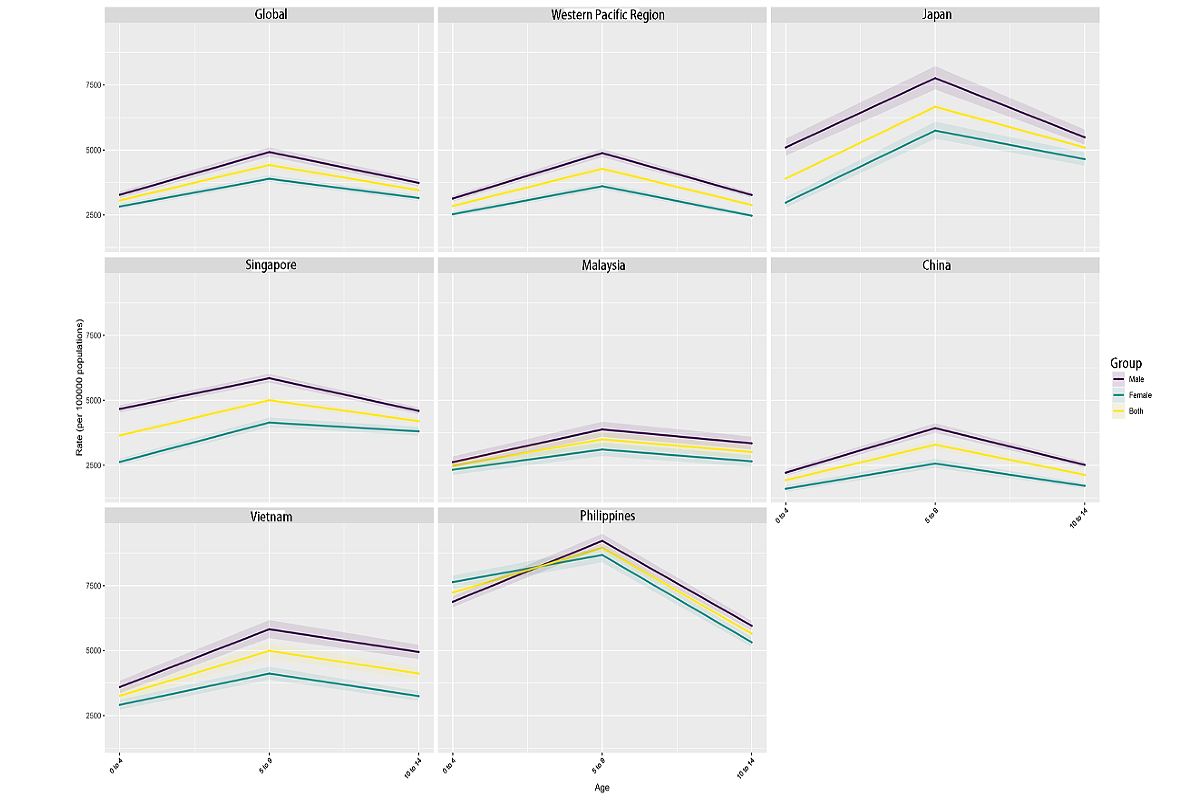
Age effects on prevalence of pediatric asthma from 1990 to 2019. This figure illustrates the fitted, longitudinal, age-specific rates for various birth cohorts, adjusted for period deviations. SDI: sociodemographic index.

The age, period, and birth cohort effects on asthma prevalence derived from the APC model are illustrated in Figure 3, Multimedia Appendix 3, and Multimedia Appendix 4, respectively. Overall, the pattern of age effects was similar across the selected regions or countries, with the lowest risk among those aged between 0 and 4 years and the risk increasing, decreasing, and then increasing with age. In addition, we found that, in all the regions, the prevalence was higher among men than among women. Overall, period effects presented an initially decreasing and then increasing risk of prevalence across different regions or countries, except in Vietnam. The Vietnam region had generally low period risks over the study period, whereas others had more unfavorable period risks most of the time. Compared with individuals in the reference period from 2000 to 2004, the relative period risk for individuals in the 2015 to 2019 period ranged from 1.25 (95% CI 1.19-1.31) in the WPR to 0.84 (95% CI 0.82-0.86) in Japan and 1.47 (95% CI 1.36-1.58) in China (Multimedia Appendix 5). Regarding birth cohort effects, there was an initially decreasing and then increasing risk of prevalence in successive birth cohorts in the WPR (Multimedia Appendix 4). Japan and Singapore (high-income countries) had favorable prevalence improvements in successive birth cohorts, whereas China, Malaysia, and Vietnam had progressive prevalence deteriorations (Multimedia Appendix 4). Compared with individuals born in the reference cohort from 1990 to 1999, the relative cohort risk for individuals born in the 2010 to 2019 cohort ranged from 1.12 (95% CI 1.06-1.20) in the WPR to 1.35 (95% CI 1.22-1.50) in China (Multimedia Appendix 6).

### Health Inequality and Future Burden

**Figure 4:**
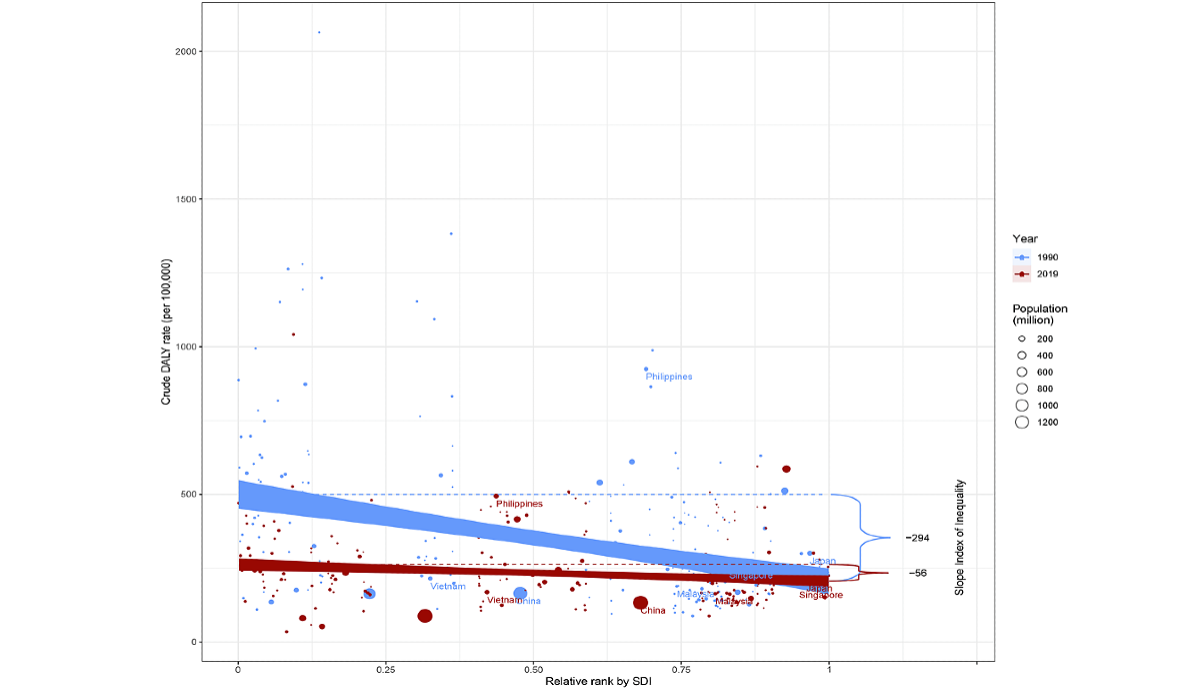
Slope index of inequality in pediatric asthma, presented using regression lines (1990 vs 2019). Regression of age-standardized disability-adjusted life year (DALY) rate on the relative rank by per capita gross domestic product. The slope index of inequality, shown as the slope of the regression line, represents the absolute difference in blindness and vision loss burden between countries or territories with the highest and lowest incomes. Dots represent countries or territories, with different sizes representing the population sizes (1990 in blue and 2019 in red). SDI: sociodemographic index.

**Figure 5:**
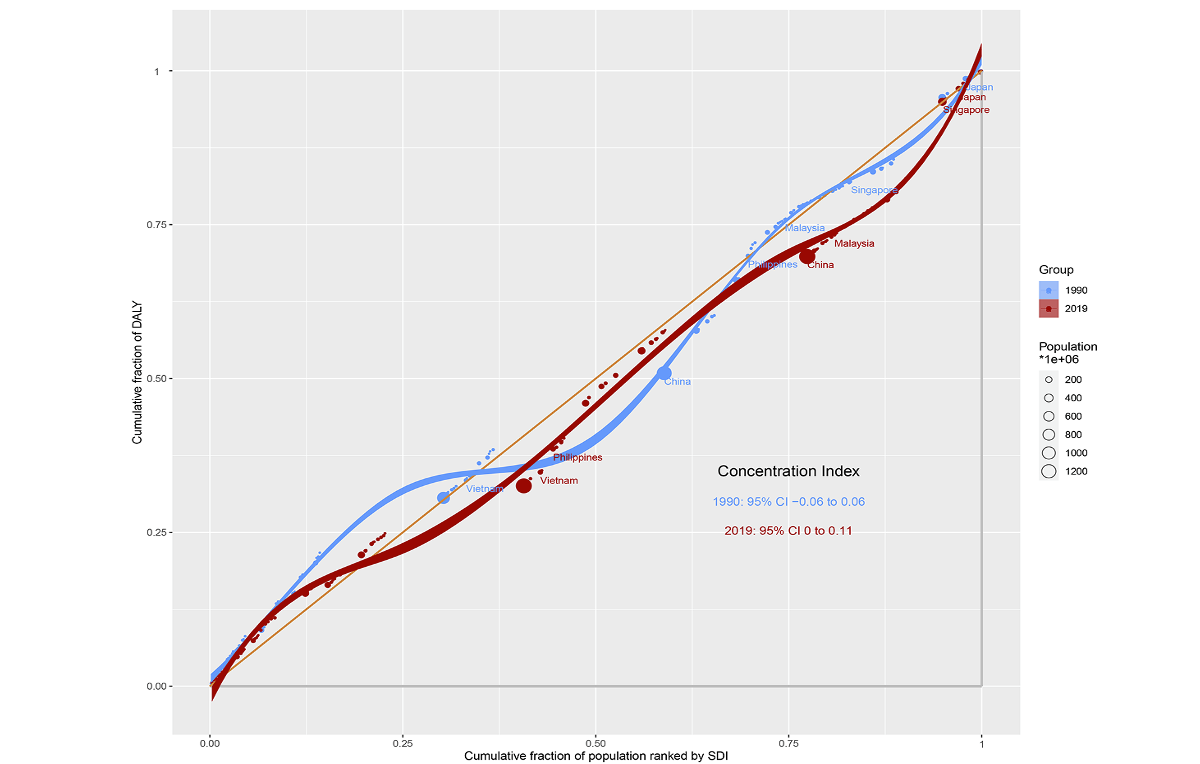
Concentration index in pediatric asthma, presented using concentration curves (1990 vs 2019). Concentration curves (Lorenz curves) graphed by plotting the cumulative fraction of population ranked by national per capita gross domestic product (x-axis) against the cumulative fraction of pediatric asthma burden ranked by age-standardized disability-adjusted life year (DALY; y-axis). The concentration index, calculated as twice the area between the 45° diagonal line and the Lorenz curve, represents the relative extent to which the pediatric asthma burden is concentrated among the poor (negative value) or the rich (positive value). Dots represent country or territories, with sizes representing population sizes (1990 in blue and 2019 in red). Trendline (in green) of the hollow rhombi demonstrates the trend in concentration index from 1990 to 2019. SDI: sociodemographic index.

The slope index of inequality was −294 and −56 DALYs per 100,000 in 1990 and 2019, respectively, showing a negative association between the age-standardized DALY rate and gross domestic product per capita. This reduction indicates that the inequality in the age-standardized burden of asthma among children between high-income and low-income countries narrowed during this time (Figure 4). In contrast, a relative inequality analysis showed that the concentration index has increased by 0.06 over 30 years, showing disproportionate concentration of the burden among the richer half of the population (Figure 5).

The projected ASPR and ASIR of childhood asthma for specific representative countries suggest an ongoing increase in both the Philippines and Japan. In contrast, the rates in other selected countries are expected to remain comparatively stable (as illustrated in Multimedia Appendices 7 and 8). In addition, DALYs associated with childhood asthma are anticipated to maintain a relatively steady trend in the future, as shown in Multimedia Appendix 9.

## Discussion

### Principal Findings

Our study used comprehensive data from the GBD Study 2019 to examine the trends in crucial childhood asthma outcomes across countries in the WPR from 1990 to 2019 and to project future patterns up to 2045. The study revealed increases in both the ASPR and ASIR, alongside decreases in mortality rates and DALYs related to childhood asthma in the WPR over the past 3 decades. This pattern persisted across nations with varying income levels, hinting at universal contributing factors. Notably, Japan showed a significant reduction in pediatric asthma DALYs, reflecting effective management strategies. According to our projections, both the Philippines and Japan might experience continued increases in the ASPR and ASIR. In summary, although the WPR is making progress in reducing fatal asthma outcomes, it continues to face increasing instances of the condition. Focusing on risk factors and ensuring equal access to health care could be key in altering these projected trends.

The gradual upward trend in the ASPR likely results from complex interactions among genetic predisposition, lifestyle changes, and environmental exposure. Key factors influencing the increasing pediatric asthma risk across nations in the WPR include increasing air pollution and urbanization, which promotes triggers such as vehicle emissions, industrial fumes, and household air pollution [24,25]; reduced rates of protective elements such as breastfeeding, which bolsters immune maturation [26]; overuse of antibiotics, linked to microbiome disruption and heightened immune reactions [27]; limited exposure to microbial diversity, particularly in urban versus rural areas [15]; and unhealthy modern lifestyles marked by poor diets, minimal exercise, and increasing obesity [26]. In addition, dietary shifts, sedentary lifestyles, and increasing obesity may contribute [28]. Hereditary factors also increase the susceptibility in Asian populations. Meanwhile, the increase in the ASIR could arise from superior diagnostic capabilities and awareness over time. Hence, although part of the escalating incidence is because of new-onset disease, improved case identification may also play a role.

Our study indicates that from 1990 to 2019, the ASPR and ASIR for pediatric asthma in countries in the WPR have notably increased, whereas the ASDR has decreased. This trend suggests that although the prevalence and incidence of asthma among children are increasing, efforts to reduce mortality after intervention measurement have been successful. These findings are consistent with those of previous studies of long-term asthma patterns. For example, a global data analysis from 1990 to 2015 showed an increase in asthma prevalence in countries in the WPR, despite a decrease in mortality rates [4]. Moreover, in Organisation for Economic Co-operation and Development countries, there has been a surge in asthma prevalence in recent decades, but child mortality rates have decreased [1]. Thus, advancements in asthma management appear to be effective in reducing fatalities among patients who have been diagnosed. However, the continual increase in asthma cases across nations points to the existing limitations in disease prevention.

This study’s per-country data offer additional insights into pediatric asthma patterns in different WPR settings. From 1990 to 2019, Australia exhibited a reduction in the ASPR while still retaining the highest ASPR, signaling scope for further progress. The substantial reduction in childhood asthma mortality and DALYs in Japan is likely owing to its cutting-edge health care system centered on universal coverage, easy access, technology integration, and personalized care [29]. This facilitates early diagnosis and evidence-based treatment. In addition, nationwide asthma control initiatives in Japan, such as the Practical Asthma Management Guideline, offer standardized diagnostic approaches and globally recognized therapies [30]. Malaysia witnessed a marginal reduction in the DALY rate; however, its burden remains higher than those of neighboring countries with comparable income levels. Thus, Malaysia may glean best practices from Japan’s robust asthma care models to enhance outcomes.

The gradual increase in the SDI has been accompanied by a corresponding increase in both the ASPR and ASIR for pediatric asthma over this study’s time frame. The SDI is indicative of socioeconomic development with factors such as income, education, and fertility rates [31,32]. It appears that pediatric populations in the WPR are still witnessing a growing burden of the disease alongside developmental indexes. Implementing targeted prevention strategies to reduce these avoidable risk factors could help in altering this trend for different socioeconomic groups.

Our age-specific analysis in the WPR reveals a decrease in asthma prevalence across various age groups, starting from <5 years and extending to older age groups This trend can be attributed to the natural maturation of the respiratory and immune systems in very young children, with approximately 75% of early-onset asthma cases resolving by midchildhood [33]. However, for those with moderate to severe asthma persisting beyond the age of 10 years, there is an increased risk of impaired lung function later in life [34]. Therefore, although prevalence decreases with age, the subset struggling with ongoing asthma beyond childhood needs adequate control and follow-up to prevent long-term pulmonary deficits.

In terms of temporal trends, the age distributions within the WPR have remained fairly stable, yet we observed fluctuating asthma risks across the decades. Following an initial reduction, there has been a recent increase in risk. This fluctuation suggests that time-related factors significantly influence asthma risk among different pediatric age groups in the region. Environmental factors are likely the primary drivers of these varying period effects. The successive birth cohorts between 2000 and 2009 exhibited escalating asthma risks compared with the preceding generation. Rapidly evolving environmental and lifestyle changes appear to influence disease susceptibility over generations differently. For example, factors such as shifting microbiome composition, increased exposure to antibiotics, and reduced number of older siblings may contribute to risk amplification in more recent cohorts [35]. In contrast, favorable cohort effects in Japan and Singapore reflect the strengths of those health systems. However, overarching regional trends highlight the need for ready-to-implement pediatric asthma strategies to break the intergenerational risk acceleration.

Our inequality assessment revealed that absolute socioeconomic disparities in childhood asthma DALYs have reduced slightly, but relative inequality persists. Low-income nations in the WPR still disproportionately face asthma burden compared with high-income economies. Barriers related to health awareness, low access to quality care, unaffordable therapies, and high out-of-pocket costs may underpin inequities. Cross-regional cooperation and localized health policy initiatives aimed at marginalized pediatric groups are required to tackle these demands.

Recent global data about pediatric asthma are limited, with most large-scale studies only presenting analyses up to 2015. Adding the latest estimates from 2016 to 2019 provides an up-to-date, 30-year perspective that is critical to capturing the current trends and trajectory changes. Forecasting future patterns up to 2045 based on recent data rather than old estimates can help anticipate potential increases or decreases in childhood asthma morbidity. This long-range projection up to 2045 is key for priority setting, resource planning, and targeted interventions by western Pacific health systems aimed at managing pediatric asthma over the next 2 decades. Specifically, updating the data up to 2019 revealed a reversal from the previous decreasing trends in some nations. These emerging increases signal to policy makers that renewed efforts are needed against pediatric asthma amid the possibly worsening patterns. The value of the 2045 projections lies in helping countries pre-empt potential resurgences and proactively formulate control strategies tailored to local risk factors. From a resource allocation perspective, the expected trajectories can direct preventive efforts and capacity building, where the impacts of pediatric asthma are likely to escalate. Overall, augmenting the data scope offers granular insights to strategically mitigate the barriers confronting children with asthma across the western Pacific area based on where the disease burdens are headed.

### Limitations

Our study had some limitations. First, it is vital to recognize the inherent constraints of GBD research. The validity and accuracy of our findings depend on the quality of the population and disease data collected, which may lead to discrepancies between our results and the actual conditions across regions. Second, the lack of comprehensive registry systems for recording mortality in many countries is another limitation. This deficiency often leads to the underestimation of actual figures. Finally, despite the multiple methodologies used in GBD studies for calculations, rectifying the disease classification errors, and reclassifying ambiguous codes, the potential inaccuracies within the data cannot be disregarded. These inherent inaccuracies can affect the reliability of our findings.

### Conclusions

Our study offers a comprehensive 30-year analysis of pediatric asthma trends in the WPR. It highlights an increase in disease prevalence alongside a decrease in mortality, amid moderate advancements in development. These trends reflect a dynamic interplay of lifestyle changes, generational transitions, obstacles in accessing health care, and demographic challenges. Addressing these issues requires focused strategies for enhancing awareness, improving prevention, ensuring equitable diagnosis and treatment, and minimizing risks. Ultimately, fostering overall population health and mitigating the risk factors are key to altering the projected trend shifts. Insights from the regional achievements in effective care models can guide future strategies.

## Appendix

Multimedia Appendix 1 Local drift and age distribution of prevalence from 1990 to 2019 for pediatric asthma. Local drift of prevalence from 1990 to 2019 for pediatric asthma. The dots and shaded areas represent the local drift (ie, annual percentage change of age-specific prevalence; percentage per year) and their corresponding 95% CIs. Temporal changes in age distribution of pediatric asthma–related disability-adjusted life years from 1990 to 2019.

Multimedia Appendix 2 Local drift of pediatric asthma prevalence in the Western Pacific Region (1990-2019).

Multimedia Appendix 3 Period effects on disability-adjusted life years for pediatric asthma. This figure illustrates the period relative risk, calculated as the ratio of age-specific rates from the 1990 to 1994 period to the 2015 to 2019 period, with the reference period set at 2000 to 2004.

Multimedia Appendix 4 Birth cohort effects on disability-adjusted life years for pediatric asthma. This figure illustrates the cohort relative risk, calculated as the ratio of age-specific rates from the 1975 to 1984 birth cohort to the 2010 to 2019 cohort, with the reference cohort set at 1990 to 1999.

Multimedia Appendix 5 Period cohort effects of pediatric asthma prevalence in the Western Pacific Region across different age groups (1990-2019).

Multimedia Appendix 6 Birth cohort effects of pediatric asthma prevalence in the Western Pacific Region (1990-2019).

Multimedia Appendix 7 Projected age-standardized prevalence rates of childhood asthma in selected representative countries from 1990 to 2045. Age-standardized prevalence rate shows ongoing increase in both the Philippines and Japan over the period.

Multimedia Appendix 8 Projected age-standardized incidence rates of childhood asthma in selected representative countries from 1990 to 2045. Age-standardized incidence rate demonstrates ongoing increase in both the Philippines and Japan over the period.

Multimedia Appendix 9 Projected disability-adjusted life years associated with childhood asthma in selected representative countries from 1990 to 2045.

## Abbreviations

APC: age-period cohort
ASDR: age-standardized death rate
ASIR: age-standardized incidence rate
ASPR: age-standardized prevalence rate
ASR: age-standardized rate
DALY: disability-adjusted life year
EAPC: estimated annual percentage change
GBD: Global Burden of Disease
SDI: sociodemographic index
WPR: Western Pacific Region

## Data Availability

The data sets generated during and analyzed during this study are available from the Global Health Data Exchange query tool on the web [12].
