# Supplementary figures and images for: Temporal Trends of Asthma Among Children in the Western Pacific Region From 1990 to 2045: Longitudinal Observational Study
Source: JMIR Public Health Surveill. 2024 Mar 14;10:e55327. doi: 10.2196/55327 (PMC10979332; doi:10.2196/55327)

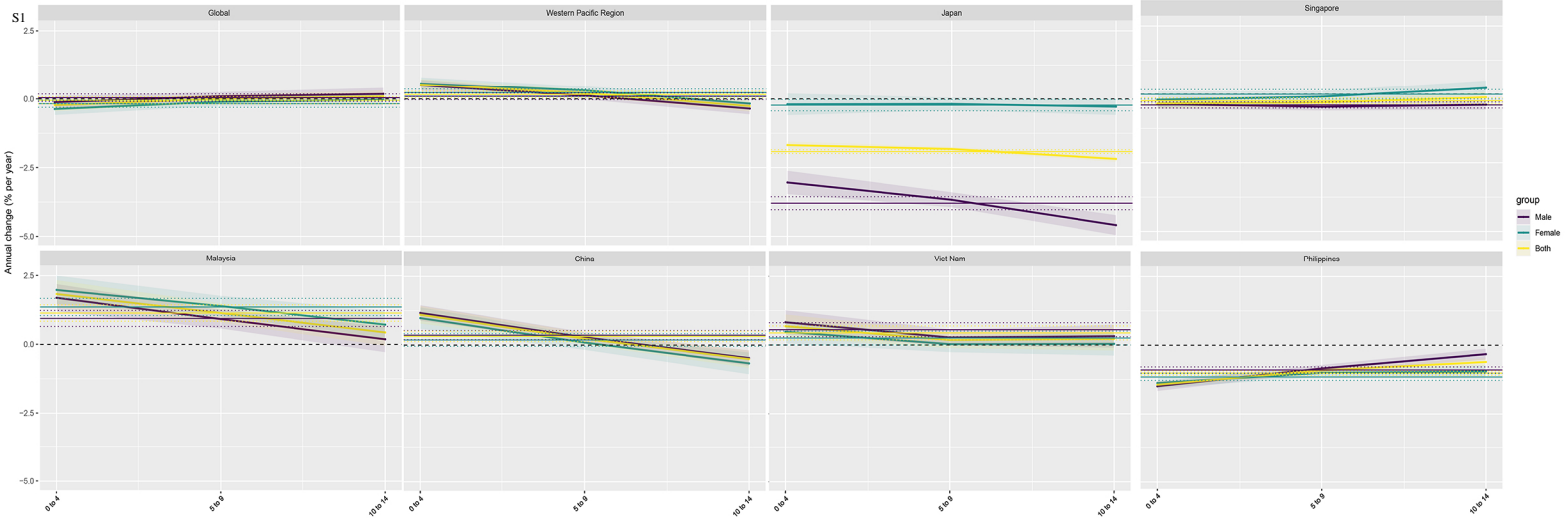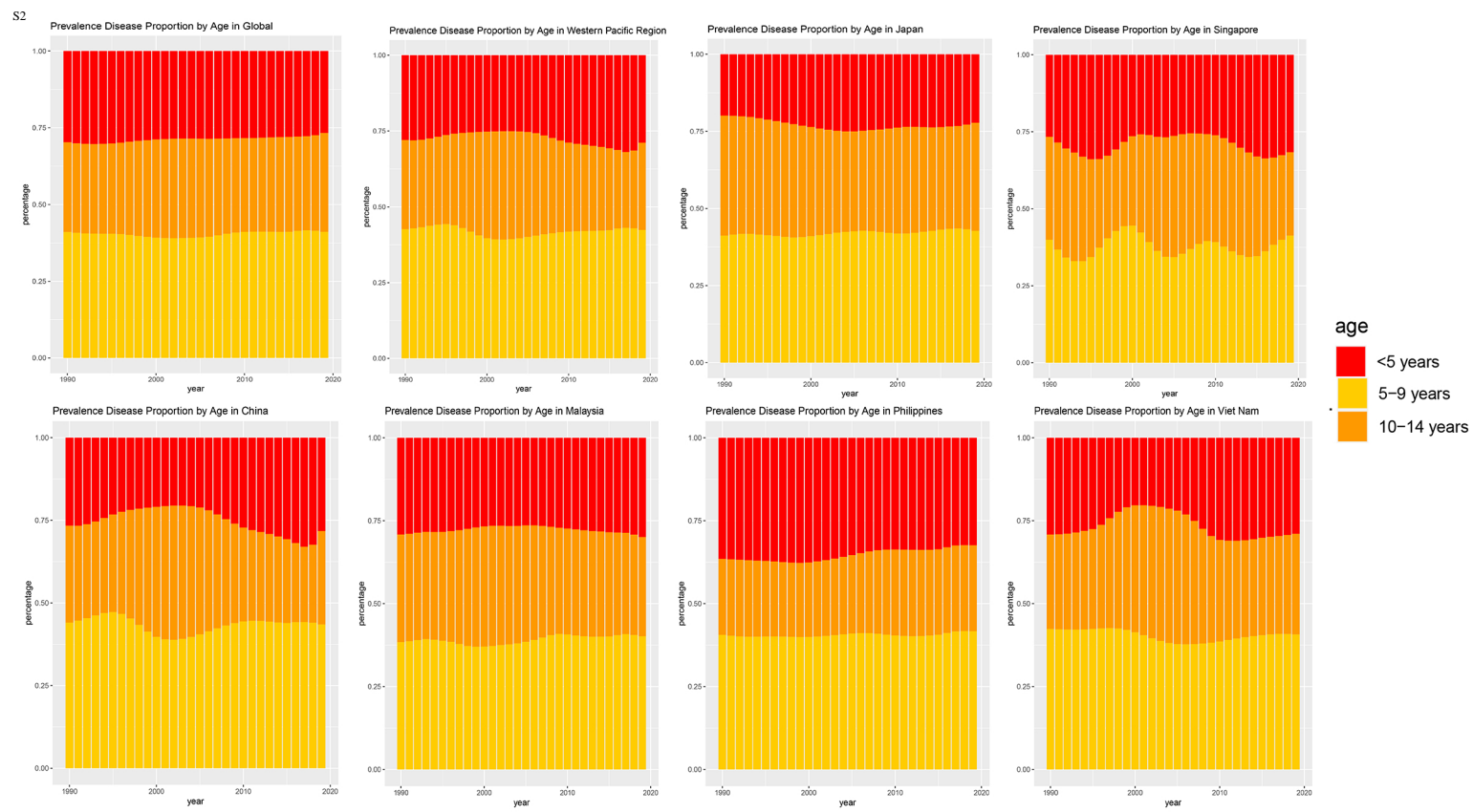

Supplement: Multimedia Appendix 1 [file publichealth_v10i1e55327_app1.pdf]

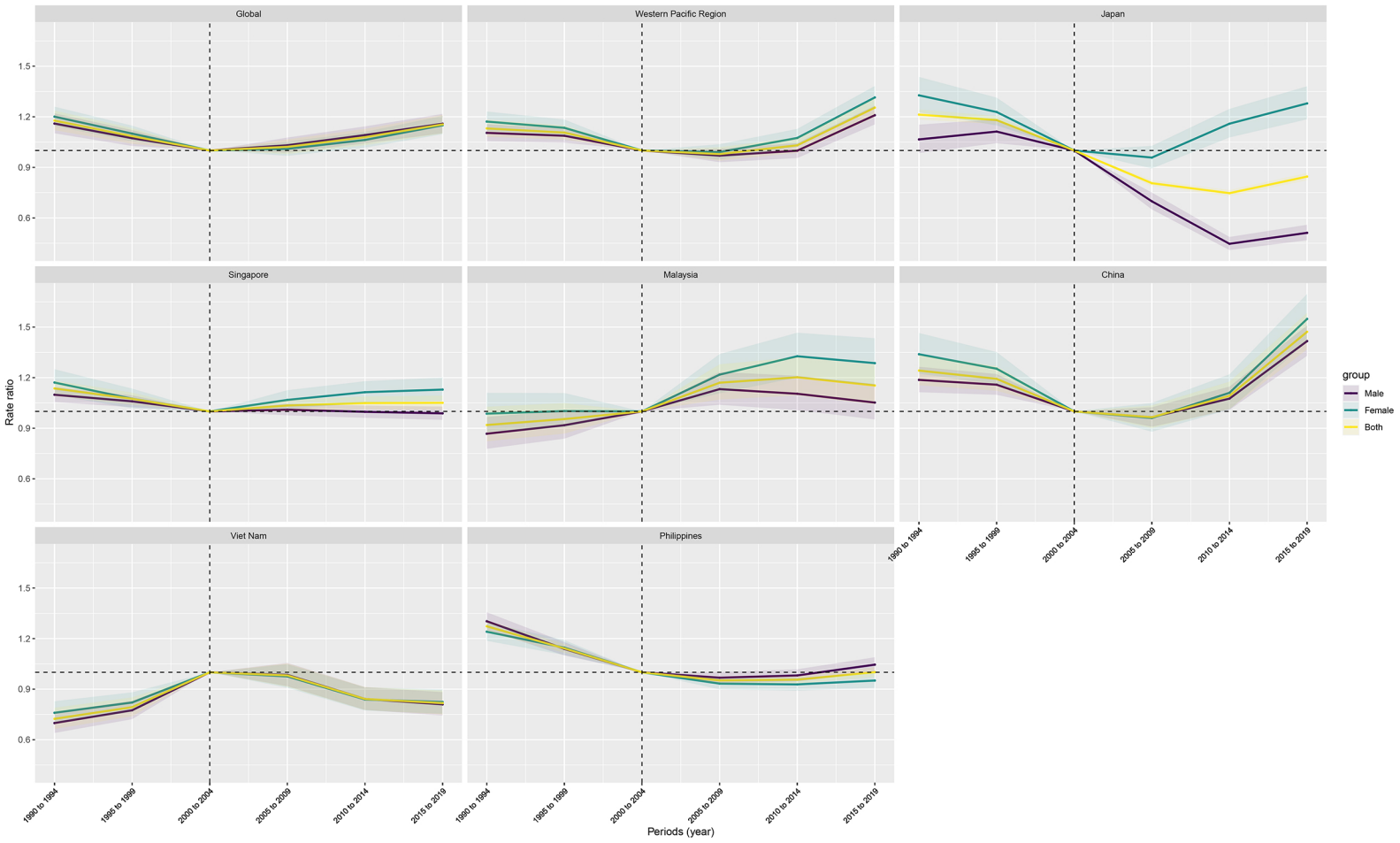

Supplement: Multimedia Appendix 3 [file publichealth_v10i1e55327_app3.pdf]

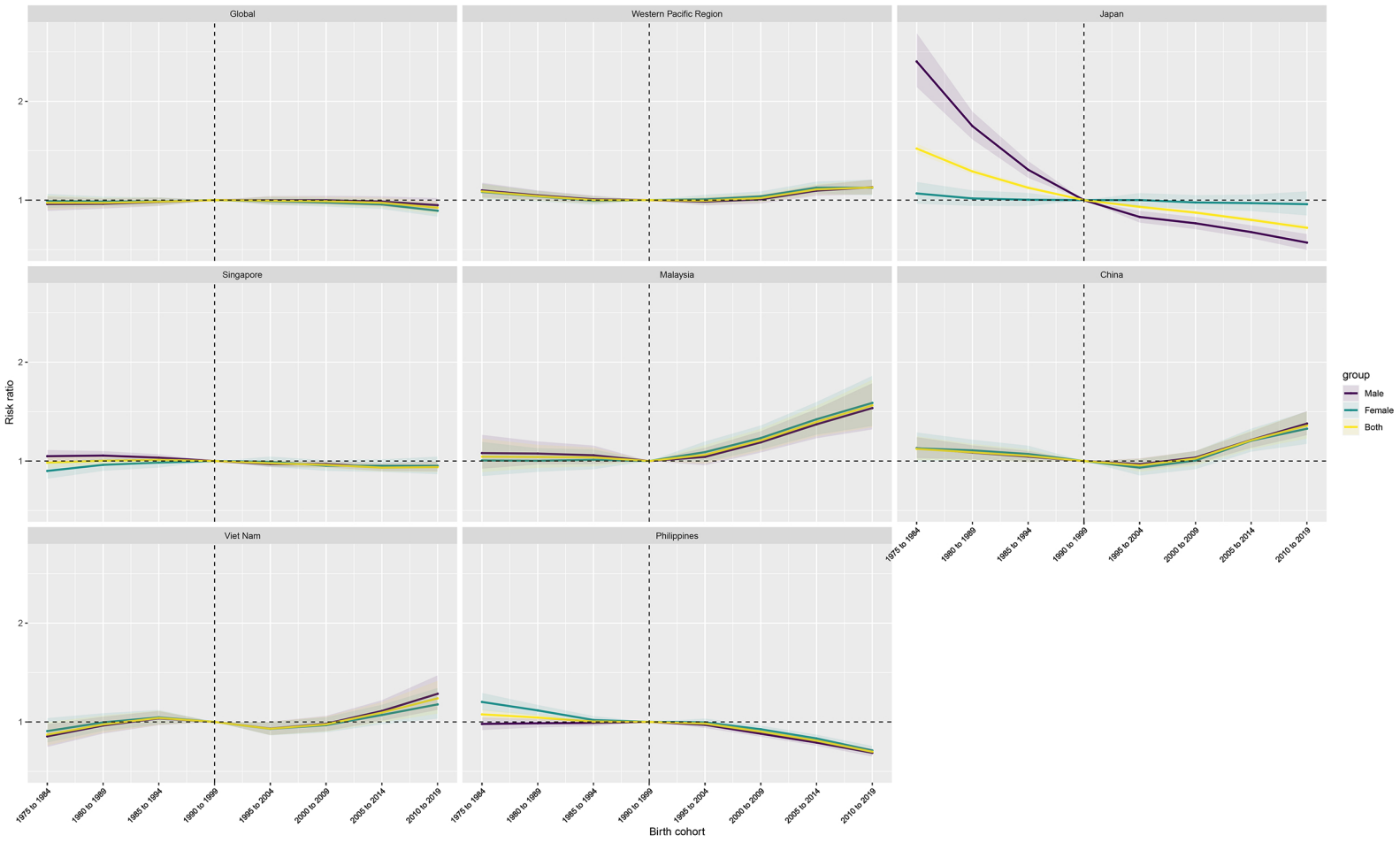

Supplement: Multimedia Appendix 4 [file publichealth_v10i1e55327_app4.pdf]

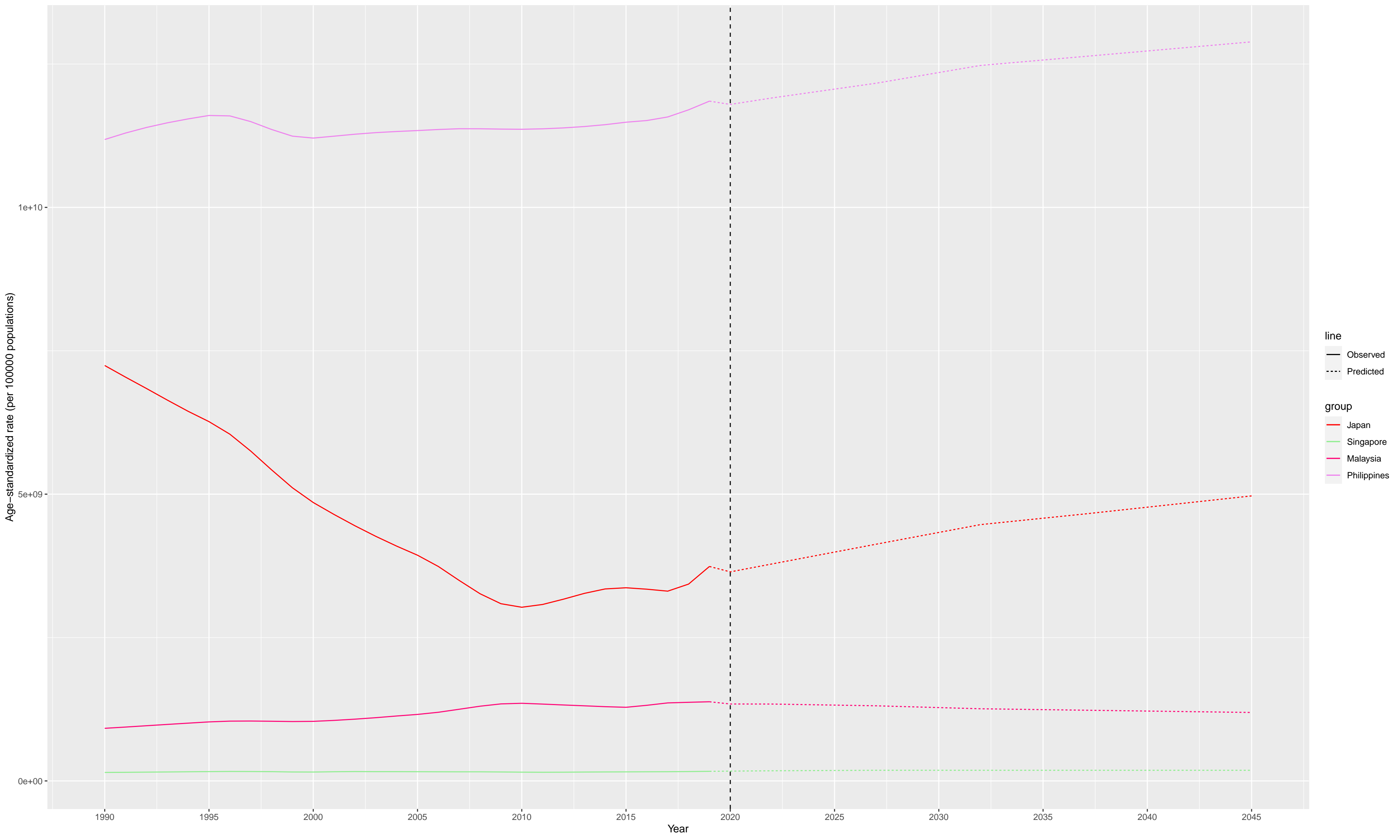

Supplement: Multimedia Appendix 7 [file publichealth_v10i1e55327_app7.pdf]

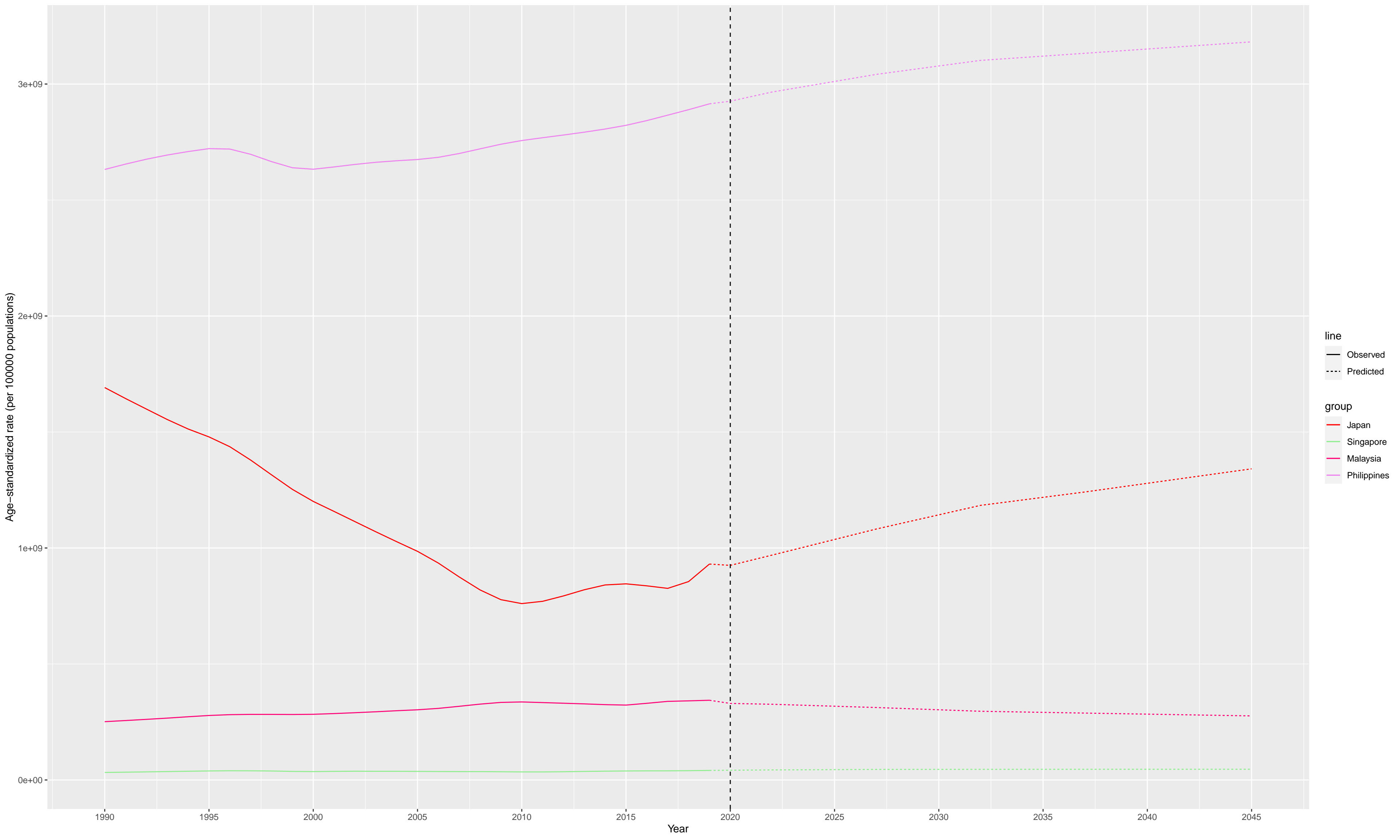

Supplement: Multimedia Appendix 8 [file publichealth_v10i1e55327_app8.pdf]

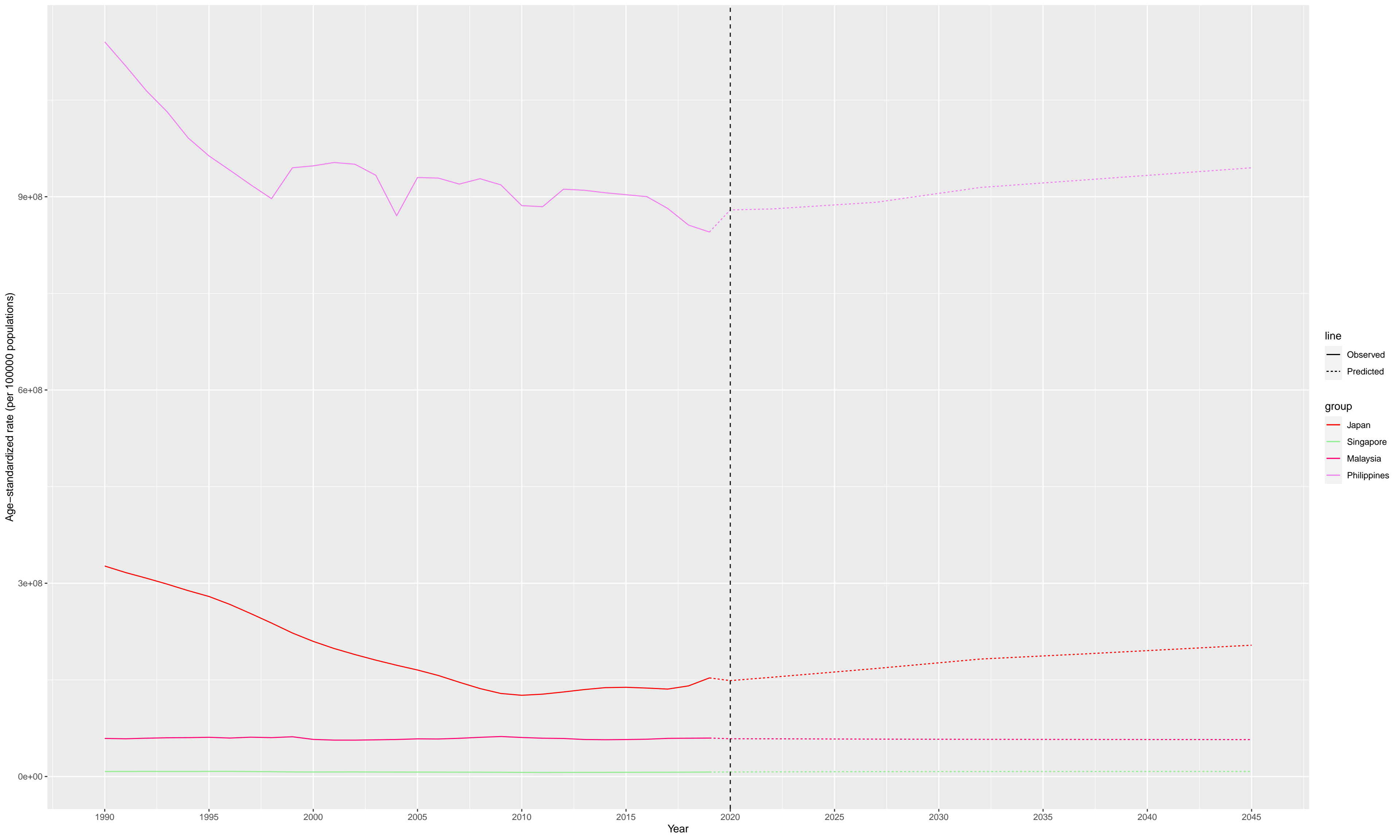

Supplement: Multimedia Appendix 9 [file publichealth_v10i1e55327_app9.pdf]
