# Supplementary material for: Temporal Trends of Asthma Among Children in the Western Pacific Region From 1990 to 2045: Longitudinal Observational Study
Source: JMIR Public Health Surveill. 2024 Mar 14;10:e55327. doi: 10.2196/55327 (PMC10979332; doi:10.2196/55327)
Supplement: Multimedia Appendix 2 [file publichealth_v10i1e55327_app2.docx]

| **Multimedia Appendix 2. Local drift of pediatric asthma prevalence in the Western Pacific Region, 1990-2019** | | | | | | | | |
| --- | --- | --- | --- | --- | --- | --- | --- | --- |
| Age | Mean Percent Change per Calendar Year | CILo | CIHi | location | cause | measure | sex | label |
| 2.5 | -0.121804532 | -0.351807897 | 0.108729716 | Global | Asthma | Prevalence | Male | 0 to 4 |
| 7.5 | 0.092465156 | -0.065814809 | 0.250995812 | Global | Asthma | Prevalence | Male | 5 to 9 |
| 12.5 | 0.180246718 | -0.047025104 | 0.408035308 | Global | Asthma | Prevalence | Male | 10 to 14 |
| 2.5 | 0.501541738 | 0.294645237 | 0.708865043 | Western Pacific Region | Asthma | Prevalence | Male | 0 to 4 |
| 7.5 | 0.121493154 | -0.019682381 | 0.262868033 | Western Pacific Region | Asthma | Prevalence | Male | 5 to 9 |
| 12.5 | -0.354115722 | -0.561361833 | -0.146437677 | Western Pacific Region | Asthma | Prevalence | Male | 10 to 14 |
| 2.5 | -3.039699774 | -3.462754109 | -2.614791491 | Japan | Asthma | Prevalence | Male | 0 to 4 |
| 7.5 | -3.669539862 | -3.94100294 | -3.397309628 | Japan | Asthma | Prevalence | Male | 5 to 9 |
| 12.5 | -4.591426713 | -4.955566312 | -4.225892001 | Japan | Asthma | Prevalence | Male | 10 to 14 |
| 2.5 | -0.365456537 | -0.53913889 | -0.191470893 | Singapore | Asthma | Prevalence | Male | 0 to 4 |
| 7.5 | -0.46442033 | -0.600030215 | -0.328625435 | Singapore | Asthma | Prevalence | Male | 5 to 9 |
| 12.5 | -0.389024717 | -0.570000861 | -0.207719172 | Singapore | Asthma | Prevalence | Male | 10 to 14 |
| 2.5 | 1.694756585 | 1.19801884 | 2.193932602 | Malaysia | Asthma | Prevalence | Male | 0 to 4 |
| 7.5 | 0.921407383 | 0.578840261 | 1.265141275 | Malaysia | Asthma | Prevalence | Male | 5 to 9 |
| 12.5 | 0.187512021 | -0.281894401 | 0.659128096 | Malaysia | Asthma | Prevalence | Male | 10 to 14 |
| 2.5 | 1.168003621 | 0.886387258 | 1.450406093 | China | Asthma | Prevalence | Male | 0 to 4 |
| 7.5 | 0.281845462 | 0.094768542 | 0.469272028 | China | Asthma | Prevalence | Male | 5 to 9 |
| 12.5 | -0.472603512 | -0.751975096 | -0.192445531 | China | Asthma | Prevalence | Male | 10 to 14 |
| 2.5 | 0.827134323 | 0.391697584 | 1.264459715 | Viet Nam | Asthma | Prevalence | Male | 0 to 4 |
| 7.5 | 0.27248205 | -0.020449594 | 0.56627196 | Viet Nam | Asthma | Prevalence | Male | 5 to 9 |
| 12.5 | 0.320209917 | -0.094951416 | 0.737096477 | Viet Nam | Asthma | Prevalence | Male | 10 to 14 |
| 2.5 | -1.489562717 | -1.662833906 | -1.315986222 | Philippines | Asthma | Prevalence | Male | 0 to 4 |
| 7.5 | -0.844964582 | -0.974049087 | -0.71571181 | Philippines | Asthma | Prevalence | Male | 5 to 9 |
| 12.5 | -0.32392458 | -0.523936171 | -0.123510835 | Philippines | Asthma | Prevalence | Male | 10 to 14 |
| 2.5 | -0.370587257 | -0.586992021 | -0.153711418 | Global | Asthma | Prevalence | Female | 0 to 4 |
| 7.5 | -0.108292054 | -0.259886918 | 0.043533219 | Global | Asthma | Prevalence | Female | 5 to 9 |
| 12.5 | -0.025199052 | -0.239004198 | 0.189064316 | Global | Asthma | Prevalence | Female | 10 to 14 |
| 2.5 | 0.575901081 | 0.353504364 | 0.798790658 | Western Pacific Region | Asthma | Prevalence | Female | 0 to 4 |
| 7.5 | 0.310516927 | 0.156391956 | 0.464879073 | Western Pacific Region | Asthma | Prevalence | Female | 5 to 9 |
| 12.5 | -0.171737458 | -0.395670712 | 0.05269925 | Western Pacific Region | Asthma | Prevalence | Female | 10 to 14 |
| 2.5 | -0.197013846 | -0.591376959 | 0.198913741 | Japan | Asthma | Prevalence | Female | 0 to 4 |
| 7.5 | -0.184126435 | -0.419499043 | 0.05180251 | Japan | Asthma | Prevalence | Female | 5 to 9 |
| 12.5 | -0.28525184 | -0.585231634 | 0.015633129 | Japan | Asthma | Prevalence | Female | 10 to 14 |
| 2.5 | -0.205269602 | -0.504626946 | 0.094988436 | Singapore | Asthma | Prevalence | Female | 0 to 4 |
| 7.5 | -0.092217466 | -0.301149461 | 0.117152373 | Singapore | Asthma | Prevalence | Female | 5 to 9 |
| 12.5 | 0.226269387 | -0.051276744 | 0.504586232 | Singapore | Asthma | Prevalence | Female | 10 to 14 |
| 2.5 | 1.980750197 | 1.461873642 | 2.502280289 | Malaysia | Asthma | Prevalence | Female | 0 to 4 |
| 7.5 | 1.395067972 | 1.025786326 | 1.76569946 | Malaysia | Asthma | Prevalence | Female | 5 to 9 |
| 12.5 | 0.719033233 | 0.212338185 | 1.22829024 | Malaysia | Asthma | Prevalence | Female | 10 to 14 |
| 2.5 | 0.980557452 | 0.592071019 | 1.370544219 | China | Asthma | Prevalence | Female | 0 to 4 |
| 7.5 | 0.092660335 | -0.171479762 | 0.35749933 | China | Asthma | Prevalence | Female | 5 to 9 |
| 12.5 | -0.664113388 | -1.058069104 | -0.268589063 | China | Asthma | Prevalence | Female | 10 to 14 |
| 2.5 | 0.479001918 | 0.061948971 | 0.89779312 | Viet Nam | Asthma | Prevalence | Female | 0 to 4 |
| 7.5 | 0.032817601 | -0.259367746 | 0.325858892 | Viet Nam | Asthma | Prevalence | Female | 5 to 9 |
| 12.5 | 0.043224561 | -0.383163432 | 0.471437615 | Viet Nam | Asthma | Prevalence | Female | 10 to 14 |
| 2.5 | -1.375252931 | -1.5618213 | -1.188330961 | Philippines | Asthma | Prevalence | Female | 0 to 4 |
| 7.5 | -1.000801056 | -1.145798721 | -0.855590711 | Philippines | Asthma | Prevalence | Female | 5 to 9 |
| 12.5 | -0.949668232 | -1.1736818 | -0.725146883 | Philippines | Asthma | Prevalence | Female | 10 to 14 |
| 2.5 | -0.231490498 | -0.453152134 | -0.009335286 | Global | Asthma | Prevalence | Both | 0 to 4 |
| 7.5 | 0.005523005 | -0.148218156 | 0.15950088 | Global | Asthma | Prevalence | Both | 5 to 9 |
| 12.5 | 0.092077788 | -0.126928224 | 0.311564047 | Global | Asthma | Prevalence | Both | 10 to 14 |
| 2.5 | 0.536740303 | 0.333984178 | 0.739906161 | Western Pacific Region | Asthma | Prevalence | Both | 0 to 4 |
| 7.5 | 0.212419902 | 0.073155334 | 0.351878274 | Western Pacific Region | Asthma | Prevalence | Both | 5 to 9 |
| 12.5 | -0.261398135 | -0.46495755 | -0.057422421 | Western Pacific Region | Asthma | Prevalence | Both | 10 to 14 |
| 2.5 | -1.681195605 | -1.808155053 | -1.554072002 | Japan | Asthma | Prevalence | Both | 0 to 4 |
| 7.5 | -1.824083664 | -1.901933151 | -1.746172396 | Japan | Asthma | Prevalence | Both | 5 to 9 |
| 12.5 | -2.185947898 | -2.287294214 | -2.084496466 | Japan | Asthma | Prevalence | Both | 10 to 14 |
| 2.5 | -0.340575676 | -0.52152555 | -0.159296657 | Singapore | Asthma | Prevalence | Both | 0 to 4 |
| 7.5 | -0.306712787 | -0.441658489 | -0.171584173 | Singapore | Asthma | Prevalence | Both | 5 to 9 |
| 12.5 | -0.114757687 | -0.29472256 | 0.065532017 | Singapore | Asthma | Prevalence | Both | 10 to 14 |
| 2.5 | 1.832640923 | 1.331159269 | 2.336604379 | Malaysia | Asthma | Prevalence | Both | 0 to 4 |
| 7.5 | 1.14158295 | 0.790610162 | 1.493777895 | Malaysia | Asthma | Prevalence | Both | 5 to 9 |
| 12.5 | 0.434877241 | -0.046427451 | 0.91849955 | Malaysia | Asthma | Prevalence | Both | 10 to 14 |
| 2.5 | 1.103277656 | 0.783763948 | 1.423804315 | China | Asthma | Prevalence | Both | 0 to 4 |
| 7.5 | 0.233737933 | 0.019500705 | 0.448434047 | China | Asthma | Prevalence | Both | 5 to 9 |
| 12.5 | -0.511024424 | -0.83075226 | -0.190265765 | China | Asthma | Prevalence | Both | 10 to 14 |
| 2.5 | 0.682817032 | 0.255086862 | 1.112372077 | Viet Nam | Asthma | Prevalence | Both | 0 to 4 |
| 7.5 | 0.18125336 | -0.111351438 | 0.474715287 | Viet Nam | Asthma | Prevalence | Both | 5 to 9 |
| 12.5 | 0.221506977 | -0.19837221 | 0.643152653 | Viet Nam | Asthma | Prevalence | Both | 10 to 14 |
| 2.5 | -1.43508231 | -1.549919062 | -1.320111608 | Philippines | Asthma | Prevalence | Both | 0 to 4 |
| 7.5 | -0.91179713 | -0.999048212 | -0.824469152 | Philippines | Asthma | Prevalence | Both | 5 to 9 |
| 12.5 | -0.608999569 | -0.744030525 | -0.473784911 | Philippines | Asthma | Prevalence | Both | 10 to 14 |
