# Supplementary material for: Temporal Trends of Asthma Among Children in the Western Pacific Region From 1990 to 2045: Longitudinal Observational Study
Source: JMIR Public Health Surveill. 2024 Mar 14;10:e55327. doi: 10.2196/55327 (PMC10979332; doi:10.2196/55327)
Supplement: Multimedia Appendix 5 [file publichealth_v10i1e55327_app5.docx]

| **Multimedia Appendix 5. Period cohort effects of pediatric asthma prevalence in the Western Pacific Region across different age groups (1990-2019).** | | | | | | | | |
| --- | --- | --- | --- | --- | --- | --- | --- | --- |
| Period | Rate Ratio | CI Lo | CI Hi | location | cause | measure | sex | label |
| 1992.5 | 1.159750521 | 1.102042781 | 1.220480089 | Global | Asthma | Prevalence | Male | 1990 to 1994 |
| 1997.5 | 1.072840361 | 1.026644177 | 1.121115247 | Global | Asthma | Prevalence | Male | 1995 to 1999 |
| 2002.5 | 1 | 1 | 1 | Global | Asthma | Prevalence | Male | 2000 to 2004 |
| 2007.5 | 1.030895793 | 0.987095478 | 1.076639655 | Global | Asthma | Prevalence | Male | 2005 to 2009 |
| 2012.5 | 1.091432141 | 1.042276361 | 1.142906203 | Global | Asthma | Prevalence | Male | 2010 to 2014 |
| 2017.5 | 1.158672729 | 1.102808415 | 1.217366929 | Global | Asthma | Prevalence | Male | 2015 to 2019 |
| 1992.5 | 1.104504145 | 1.055304552 | 1.155997483 | Western Pacific Region | Asthma | Prevalence | Male | 1990 to 1994 |
| 1997.5 | 1.087492116 | 1.046450389 | 1.130143497 | Western Pacific Region | Asthma | Prevalence | Male | 1995 to 1999 |
| 2002.5 | 1 | 1 | 1 | Western Pacific Region | Asthma | Prevalence | Male | 2000 to 2004 |
| 2007.5 | 0.969520029 | 0.930428054 | 1.010254454 | Western Pacific Region | Asthma | Prevalence | Male | 2005 to 2009 |
| 2012.5 | 0.998667969 | 0.955078299 | 1.044247066 | Western Pacific Region | Asthma | Prevalence | Male | 2010 to 2014 |
| 2017.5 | 1.209325918 | 1.154854743 | 1.266366343 | Western Pacific Region | Asthma | Prevalence | Male | 2015 to 2019 |
| 1992.5 | 1.065675145 | 0.984976119 | 1.152985837 | Japan | Asthma | Prevalence | Male | 1990 to 1994 |
| 1997.5 | 1.112240608 | 1.043097825 | 1.18596659 | Japan | Asthma | Prevalence | Male | 1995 to 1999 |
| 2002.5 | 1 | 1 | 1 | Japan | Asthma | Prevalence | Male | 2000 to 2004 |
| 2007.5 | 0.698398258 | 0.650157516 | 0.750218392 | Japan | Asthma | Prevalence | Male | 2005 to 2009 |
| 2012.5 | 0.446364647 | 0.409308535 | 0.486775576 | Japan | Asthma | Prevalence | Male | 2010 to 2014 |
| 2017.5 | 0.511452099 | 0.467758901 | 0.559226663 | Japan | Asthma | Prevalence | Male | 2015 to 2019 |
| 1992.5 | 1.09877415 | 1.055750807 | 1.143550755 | Singapore | Asthma | Prevalence | Male | 1990 to 1994 |
| 1997.5 | 1.059543708 | 1.024735967 | 1.095533781 | Singapore | Asthma | Prevalence | Male | 1995 to 1999 |
| 2002.5 | 1 | 1 | 1 | Singapore | Asthma | Prevalence | Male | 2000 to 2004 |
| 2007.5 | 1.010088803 | 0.976681323 | 1.044638988 | Singapore | Asthma | Prevalence | Male | 2005 to 2009 |
| 2012.5 | 0.997492351 | 0.961910887 | 1.034389988 | Singapore | Asthma | Prevalence | Male | 2010 to 2014 |
| 2017.5 | 0.988393978 | 0.949770701 | 1.028587905 | Singapore | Asthma | Prevalence | Male | 2015 to 2019 |
| 1992.5 | 0.867431747 | 0.778275178 | 0.966801791 | Malaysia | Asthma | Prevalence | Male | 1990 to 1994 |
| 1997.5 | 0.918008732 | 0.838094478 | 1.005542995 | Malaysia | Asthma | Prevalence | Male | 1995 to 1999 |
| 2002.5 | 1 | 1 | 1 | Malaysia | Asthma | Prevalence | Male | 2000 to 2004 |
| 2007.5 | 1.132185705 | 1.039086112 | 1.233626796 | Malaysia | Asthma | Prevalence | Male | 2005 to 2009 |
| 2012.5 | 1.104568417 | 1.007461566 | 1.211035168 | Malaysia | Asthma | Prevalence | Male | 2010 to 2014 |
| 2017.5 | 1.052288251 | 0.952411048 | 1.16263935 | Malaysia | Asthma | Prevalence | Male | 2015 to 2019 |
| 1992.5 | 1.186875569 | 1.113806864 | 1.264737776 | China | Asthma | Prevalence | Male | 1990 to 1994 |
| 1997.5 | 1.158398884 | 1.098483807 | 1.221581934 | China | Asthma | Prevalence | Male | 1995 to 1999 |
| 2002.5 | 1 | 1 | 1 | China | Asthma | Prevalence | Male | 2000 to 2004 |
| 2007.5 | 0.965039806 | 0.909005628 | 1.024528121 | China | Asthma | Prevalence | Male | 2005 to 2009 |
| 2012.5 | 1.075725531 | 1.008989255 | 1.146875859 | China | Asthma | Prevalence | Male | 2010 to 2014 |
| 2017.5 | 1.417000722 | 1.329696212 | 1.510037426 | China | Asthma | Prevalence | Male | 2015 to 2019 |
| 1992.5 | 0.698789981 | 0.640114473 | 0.762843926 | Viet Nam | Asthma | Prevalence | Male | 1990 to 1994 |
| 1997.5 | 0.774612044 | 0.721661329 | 0.831447931 | Viet Nam | Asthma | Prevalence | Male | 1995 to 1999 |
| 2002.5 | 1 | 1 | 1 | Viet Nam | Asthma | Prevalence | Male | 2000 to 2004 |
| 2007.5 | 0.984480037 | 0.917821984 | 1.055979219 | Viet Nam | Asthma | Prevalence | Male | 2005 to 2009 |
| 2012.5 | 0.841729171 | 0.776809334 | 0.912074517 | Viet Nam | Asthma | Prevalence | Male | 2010 to 2014 |
| 2017.5 | 0.809802238 | 0.743403882 | 0.882131073 | Viet Nam | Asthma | Prevalence | Male | 2015 to 2019 |
| 1992.5 | 1.302777302 | 1.251258828 | 1.356416962 | Philippines | Asthma | Prevalence | Male | 1990 to 1994 |
| 1997.5 | 1.138882727 | 1.099901665 | 1.179245297 | Philippines | Asthma | Prevalence | Male | 1995 to 1999 |
| 2002.5 | 1 | 1 | 1 | Philippines | Asthma | Prevalence | Male | 2000 to 2004 |
| 2007.5 | 0.967764231 | 0.935453585 | 1.00119089 | Philippines | Asthma | Prevalence | Male | 2005 to 2009 |
| 2012.5 | 0.981057323 | 0.945455844 | 1.017999388 | Philippines | Asthma | Prevalence | Male | 2010 to 2014 |
| 2017.5 | 1.045297116 | 1.003338079 | 1.089010855 | Philippines | Asthma | Prevalence | Male | 2015 to 2019 |
| 1992.5 | 1.200691439 | 1.14417504 | 1.25999946 | Global | Asthma | Prevalence | Female | 1990 to 1994 |
| 1997.5 | 1.100238215 | 1.055010606 | 1.147404701 | Global | Asthma | Prevalence | Female | 1995 to 1999 |
| 2002.5 | 1 | 1 | 1 | Global | Asthma | Prevalence | Female | 2000 to 2004 |
| 2007.5 | 1.009587212 | 0.968276466 | 1.052660449 | Global | Asthma | Prevalence | Female | 2005 to 2009 |
| 2012.5 | 1.062494108 | 1.016680784 | 1.110371857 | Global | Asthma | Prevalence | Female | 2010 to 2014 |
| 2017.5 | 1.149812717 | 1.096810099 | 1.205376651 | Global | Asthma | Prevalence | Female | 2015 to 2019 |
| 1992.5 | 1.171491337 | 1.114697918 | 1.231178359 | Western Pacific Region | Asthma | Prevalence | Female | 1990 to 1994 |
| 1997.5 | 1.134573717 | 1.087277635 | 1.183927157 | Western Pacific Region | Asthma | Prevalence | Female | 1995 to 1999 |
| 2002.5 | 1 | 1 | 1 | Western Pacific Region | Asthma | Prevalence | Female | 2000 to 2004 |
| 2007.5 | 0.990701721 | 0.9462495 | 1.037242186 | Western Pacific Region | Asthma | Prevalence | Female | 2005 to 2009 |
| 2012.5 | 1.074137415 | 1.022850689 | 1.127995707 | Western Pacific Region | Asthma | Prevalence | Female | 2010 to 2014 |
| 2017.5 | 1.314671936 | 1.249839504 | 1.382867394 | Western Pacific Region | Asthma | Prevalence | Female | 2015 to 2019 |
| 1992.5 | 1.327256285 | 1.226478843 | 1.436314419 | Japan | Asthma | Prevalence | Female | 1990 to 1994 |
| 1997.5 | 1.228360051 | 1.148574148 | 1.313688295 | Japan | Asthma | Prevalence | Female | 1995 to 1999 |
| 2002.5 | 1 | 1 | 1 | Japan | Asthma | Prevalence | Female | 2000 to 2004 |
| 2007.5 | 0.957918825 | 0.893128923 | 1.027408756 | Japan | Asthma | Prevalence | Female | 2005 to 2009 |
| 2012.5 | 1.158960615 | 1.077459729 | 1.246626366 | Japan | Asthma | Prevalence | Female | 2010 to 2014 |
| 2017.5 | 1.27940049 | 1.185087803 | 1.381218851 | Japan | Asthma | Prevalence | Female | 2015 to 2019 |
| 1992.5 | 1.171226154 | 1.097315982 | 1.250114576 | Singapore | Asthma | Prevalence | Female | 1990 to 1994 |
| 1997.5 | 1.075068795 | 1.018185919 | 1.13512954 | Singapore | Asthma | Prevalence | Female | 1995 to 1999 |
| 2002.5 | 1 | 1 | 1 | Singapore | Asthma | Prevalence | Female | 2000 to 2004 |
| 2007.5 | 1.067965108 | 1.013270197 | 1.125612374 | Singapore | Asthma | Prevalence | Female | 2005 to 2009 |
| 2012.5 | 1.11385156 | 1.052158833 | 1.179161605 | Singapore | Asthma | Prevalence | Female | 2010 to 2014 |
| 2017.5 | 1.129401178 | 1.061982494 | 1.201099856 | Singapore | Asthma | Prevalence | Female | 2015 to 2019 |
| 1992.5 | 0.986574302 | 0.877009445 | 1.109827105 | Malaysia | Asthma | Prevalence | Female | 1990 to 1994 |
| 1997.5 | 1.001934402 | 0.90524509 | 1.108951109 | Malaysia | Asthma | Prevalence | Female | 1995 to 1999 |
| 2002.5 | 1 | 1 | 1 | Malaysia | Asthma | Prevalence | Female | 2000 to 2004 |
| 2007.5 | 1.218015728 | 1.107030609 | 1.340127637 | Malaysia | Asthma | Prevalence | Female | 2005 to 2009 |
| 2012.5 | 1.327134454 | 1.201038543 | 1.466469057 | Malaysia | Asthma | Prevalence | Female | 2010 to 2014 |
| 2017.5 | 1.286227147 | 1.153654153 | 1.434034861 | Malaysia | Asthma | Prevalence | Female | 2015 to 2019 |
| 1992.5 | 1.338908463 | 1.223690299 | 1.464975144 | China | Asthma | Prevalence | Female | 1990 to 1994 |
| 1997.5 | 1.253133343 | 1.161460412 | 1.352041928 | China | Asthma | Prevalence | Female | 1995 to 1999 |
| 2002.5 | 1 | 1 | 1 | China | Asthma | Prevalence | Female | 2000 to 2004 |
| 2007.5 | 0.960963967 | 0.879437076 | 1.050048687 | China | Asthma | Prevalence | Female | 2005 to 2009 |
| 2012.5 | 1.110396764 | 1.010791443 | 1.219817384 | China | Asthma | Prevalence | Female | 2010 to 2014 |
| 2017.5 | 1.548563813 | 1.413029302 | 1.697098482 | China | Asthma | Prevalence | Female | 2015 to 2019 |
| 1992.5 | 0.759788273 | 0.697146138 | 0.828059123 | Viet Nam | Asthma | Prevalence | Female | 1990 to 1994 |
| 1997.5 | 0.820764873 | 0.764676864 | 0.880966861 | Viet Nam | Asthma | Prevalence | Female | 1995 to 1999 |
| 2002.5 | 1 | 1 | 1 | Viet Nam | Asthma | Prevalence | Female | 2000 to 2004 |
| 2007.5 | 0.975665506 | 0.908416323 | 1.04789308 | Viet Nam | Asthma | Prevalence | Female | 2005 to 2009 |
| 2012.5 | 0.838657189 | 0.772885858 | 0.910025555 | Viet Nam | Asthma | Prevalence | Female | 2010 to 2014 |
| 2017.5 | 0.82371654 | 0.754568998 | 0.899200657 | Viet Nam | Asthma | Prevalence | Female | 2015 to 2019 |
| 1992.5 | 1.240906248 | 1.1867676 | 1.297514625 | Philippines | Asthma | Prevalence | Female | 1990 to 1994 |
| 1997.5 | 1.146136475 | 1.102627788 | 1.191361975 | Philippines | Asthma | Prevalence | Female | 1995 to 1999 |
| 2002.5 | 1 | 1 | 1 | Philippines | Asthma | Prevalence | Female | 2000 to 2004 |
| 2007.5 | 0.93293604 | 0.898097739 | 0.969125761 | Philippines | Asthma | Prevalence | Female | 2005 to 2009 |
| 2012.5 | 0.928175198 | 0.890542968 | 0.967397678 | Philippines | Asthma | Prevalence | Female | 2010 to 2014 |
| 2017.5 | 0.951045282 | 0.907843589 | 0.996302821 | Philippines | Asthma | Prevalence | Female | 2015 to 2019 |
| 1992.5 | 1.177270238 | 1.120669136 | 1.236730064 | Global | Asthma | Prevalence | Both | 1990 to 1994 |
| 1997.5 | 1.084785303 | 1.039473272 | 1.132072547 | Global | Asthma | Prevalence | Both | 1995 to 1999 |
| 2002.5 | 1 | 1 | 1 | Global | Asthma | Prevalence | Both | 2000 to 2004 |
| 2007.5 | 1.021559996 | 0.979294932 | 1.065649164 | Global | Asthma | Prevalence | Both | 2005 to 2009 |
| 2012.5 | 1.078797815 | 1.031604371 | 1.128150247 | Global | Asthma | Prevalence | Both | 2010 to 2014 |
| 2017.5 | 1.154771478 | 1.100724252 | 1.211472505 | Global | Asthma | Prevalence | Both | 2015 to 2019 |
| 1992.5 | 1.130766799 | 1.081101194 | 1.182714033 | Western Pacific Region | Asthma | Prevalence | Both | 1990 to 1994 |
| 1997.5 | 1.106093069 | 1.064685033 | 1.149111558 | Western Pacific Region | Asthma | Prevalence | Both | 1995 to 1999 |
| 2002.5 | 1 | 1 | 1 | Western Pacific Region | Asthma | Prevalence | Both | 2000 to 2004 |
| 2007.5 | 0.978741871 | 0.939470283 | 1.019655084 | Western Pacific Region | Asthma | Prevalence | Both | 2005 to 2009 |
| 2012.5 | 1.030600186 | 0.986148102 | 1.077056014 | Western Pacific Region | Asthma | Prevalence | Both | 2010 to 2014 |
| 2017.5 | 1.25416899 | 1.198326895 | 1.31261333 | Western Pacific Region | Asthma | Prevalence | Both | 2015 to 2019 |
| 1992.5 | 1.212672982 | 1.18341527 | 1.242654035 | Japan | Asthma | Prevalence | Both | 1990 to 1994 |
| 1997.5 | 1.180403813 | 1.156540625 | 1.204759376 | Japan | Asthma | Prevalence | Both | 1995 to 1999 |
| 2002.5 | 1 | 1 | 1 | Japan | Asthma | Prevalence | Both | 2000 to 2004 |
| 2007.5 | 0.805947483 | 0.78839146 | 0.823894446 | Japan | Asthma | Prevalence | Both | 2005 to 2009 |
| 2012.5 | 0.746757042 | 0.728923357 | 0.765027042 | Japan | Asthma | Prevalence | Both | 2010 to 2014 |
| 2017.5 | 0.84521804 | 0.82404753 | 0.86693244 | Japan | Asthma | Prevalence | Both | 2015 to 2019 |
| 1992.5 | 1.135988696 | 1.090700775 | 1.183157055 | Singapore | Asthma | Prevalence | Both | 1990 to 1994 |
| 1997.5 | 1.074454787 | 1.038519827 | 1.111633173 | Singapore | Asthma | Prevalence | Both | 1995 to 1999 |
| 2002.5 | 1 | 1 | 1 | Singapore | Asthma | Prevalence | Both | 2000 to 2004 |
| 2007.5 | 1.035034234 | 1.000782266 | 1.070458483 | Singapore | Asthma | Prevalence | Both | 2005 to 2009 |
| 2012.5 | 1.049283024 | 1.01176384 | 1.08819353 | Singapore | Asthma | Prevalence | Both | 2010 to 2014 |
| 2017.5 | 1.05071481 | 1.009819068 | 1.093266752 | Singapore | Asthma | Prevalence | Both | 2015 to 2019 |
| 1992.5 | 0.919193815 | 0.822269635 | 1.027542832 | Malaysia | Asthma | Prevalence | Both | 1990 to 1994 |
| 1997.5 | 0.954369974 | 0.86811619 | 1.049193712 | Malaysia | Asthma | Prevalence | Both | 1995 to 1999 |
| 2002.5 | 1 | 1 | 1 | Malaysia | Asthma | Prevalence | Both | 2000 to 2004 |
| 2007.5 | 1.170056161 | 1.070166604 | 1.279269429 | Malaysia | Asthma | Prevalence | Both | 2005 to 2009 |
| 2012.5 | 1.202346014 | 1.093913972 | 1.321526165 | Malaysia | Asthma | Prevalence | Both | 2010 to 2014 |
| 2017.5 | 1.153531898 | 1.040971279 | 1.278263741 | Malaysia | Asthma | Prevalence | Both | 2015 to 2019 |
| 1992.5 | 1.242134196 | 1.154925082 | 1.335928524 | China | Asthma | Prevalence | Both | 1990 to 1994 |
| 1997.5 | 1.192976229 | 1.122262251 | 1.268145909 | China | Asthma | Prevalence | Both | 1995 to 1999 |
| 2002.5 | 1 | 1 | 1 | China | Asthma | Prevalence | Both | 2000 to 2004 |
| 2007.5 | 0.965373773 | 0.900310929 | 1.035138518 | China | Asthma | Prevalence | Both | 2005 to 2009 |
| 2012.5 | 1.092237225 | 1.013897622 | 1.1766298 | China | Asthma | Prevalence | Both | 2010 to 2014 |
| 2017.5 | 1.472176568 | 1.368035314 | 1.584245542 | China | Asthma | Prevalence | Both | 2015 to 2019 |
| 1992.5 | 0.723803636 | 0.66351427 | 0.789571118 | Viet Nam | Asthma | Prevalence | Both | 1990 to 1994 |
| 1997.5 | 0.79300015 | 0.738802595 | 0.851173565 | Viet Nam | Asthma | Prevalence | Both | 1995 to 1999 |
| 2002.5 | 1 | 1 | 1 | Viet Nam | Asthma | Prevalence | Both | 2000 to 2004 |
| 2007.5 | 0.981530702 | 0.914577995 | 1.053384756 | Viet Nam | Asthma | Prevalence | Both | 2005 to 2009 |
| 2012.5 | 0.841678662 | 0.776320158 | 0.912539708 | Viet Nam | Asthma | Prevalence | Both | 2010 to 2014 |
| 2017.5 | 0.817307918 | 0.749648779 | 0.891073595 | Viet Nam | Asthma | Prevalence | Both | 2015 to 2019 |
| 1992.5 | 1.272894761 | 1.238909943 | 1.307811823 | Philippines | Asthma | Prevalence | Both | 1990 to 1994 |
| 1997.5 | 1.141847759 | 1.115421088 | 1.168900533 | Philippines | Asthma | Prevalence | Both | 1995 to 1999 |
| 2002.5 | 1 | 1 | 1 | Philippines | Asthma | Prevalence | Both | 2000 to 2004 |
| 2007.5 | 0.951648325 | 0.930086956 | 0.973709533 | Philippines | Asthma | Prevalence | Both | 2005 to 2009 |
| 2012.5 | 0.956286413 | 0.932738582 | 0.980428731 | Philippines | Asthma | Prevalence | Both | 2010 to 2014 |
| 2017.5 | 1.000829074 | 0.973378524 | 1.029053766 | Philippines | Asthma | Prevalence | Both | 2015 to 2019 |
