# Supplementary material for: Temporal Trends of Asthma Among Children in the Western Pacific Region From 1990 to 2045: Longitudinal Observational Study
Source: JMIR Public Health Surveill. 2024 Mar 14;10:e55327. doi: 10.2196/55327 (PMC10979332; doi:10.2196/55327)
Supplement: Multimedia Appendix 6 [file publichealth_v10i1e55327_app6.docx]

| **Multimedia Appendix 6. Birth cohort effects of pediatric asthma prevalence in the Western Pacific Region (1990-2019).** | | | | | | |
| --- | --- | --- | --- | --- | --- | --- |
| **Cohort** | **Rate Ratio** | **CILo** | **CIHi** | **location** | **sex** | **label** |
| 1980 | 0.960676875 | 0.890338887 | 1.03657166 | Global | Male | 1975 to 1984 |
| 1985 | 0.963352071 | 0.913789881 | 1.015602418 | Global | Male | 1980 to 1989 |
| 1990 | 0.981842525 | 0.940183496 | 1.025347443 | Global | Male | 1985 to 1994 |
| 1995 | 1 | 1 | 1 | Global | Male | 1990 to 1999 |
| 2000 | 0.997992671 | 0.95599598 | 1.041834268 | Global | Male | 1995 to 2004 |
| 2005 | 0.998053234 | 0.954260363 | 1.043855844 | Global | Male | 2000 to 2009 |
| 2010 | 0.985691332 | 0.935401772 | 1.038684586 | Global | Male | 2005 to 2014 |
| 2015 | 0.94899966 | 0.881998864 | 1.021090153 | Global | Male | 2010 to 2019 |
| 1980 | 1.098460799 | 1.026179493 | 1.175833405 | Western Pacific Region | Male | 1975 to 1984 |
| 1985 | 1.04818524 | 0.999959011 | 1.098737334 | Western Pacific Region | Male | 1980 to 1989 |
| 1990 | 1.007342417 | 0.969501359 | 1.046660467 | Western Pacific Region | Male | 1985 to 1994 |
| 1995 | 1 | 1 | 1 | Western Pacific Region | Male | 1990 to 1999 |
| 2000 | 0.985650464 | 0.945996778 | 1.026966328 | Western Pacific Region | Male | 1995 to 2004 |
| 2005 | 1.008153927 | 0.965397482 | 1.052804011 | Western Pacific Region | Male | 2000 to 2009 |
| 2010 | 1.096326071 | 1.04428602 | 1.150959441 | Western Pacific Region | Male | 2005 to 2014 |
| 2015 | 1.130570986 | 1.05883404 | 1.207168175 | Western Pacific Region | Male | 2010 to 2019 |
| 1980 | 2.402932086 | 2.145473886 | 2.691285431 | Japan | Male | 1975 to 1984 |
| 1985 | 1.749148996 | 1.613896466 | 1.895736359 | Japan | Male | 1980 to 1989 |
| 1990 | 1.307370255 | 1.224813216 | 1.395491951 | Japan | Male | 1985 to 1994 |
| 1995 | 1 | 1 | 1 | Japan | Male | 1990 to 1999 |
| 2000 | 0.829563051 | 0.772064193 | 0.891344089 | Japan | Male | 1995 to 2004 |
| 2005 | 0.765537933 | 0.706358053 | 0.829676003 | Japan | Male | 2000 to 2009 |
| 2010 | 0.676461794 | 0.614602704 | 0.744546933 | Japan | Male | 2005 to 2014 |
| 2015 | 0.570187218 | 0.49486327 | 0.656976348 | Japan | Male | 2010 to 2019 |
| 1980 | 1.04887489 | 0.989502997 | 1.111809199 | Singapore | Male | 1975 to 1984 |
| 1985 | 1.055063646 | 1.011112885 | 1.100924847 | Singapore | Male | 1980 to 1989 |
| 1990 | 1.033752093 | 1.000307628 | 1.068314746 | Singapore | Male | 1985 to 1994 |
| 1995 | 1 | 1 | 1 | Singapore | Male | 1990 to 1999 |
| 2000 | 0.969622812 | 0.937802416 | 1.0025229 | Singapore | Male | 1995 to 2004 |
| 2005 | 0.969090193 | 0.935493259 | 1.003893715 | Singapore | Male | 2000 to 2009 |
| 2010 | 0.937630372 | 0.899557941 | 0.97731416 | Singapore | Male | 2005 to 2014 |
| 2015 | 0.945350499 | 0.894997228 | 0.998536685 | Singapore | Male | 2010 to 2019 |
| 1980 | 1.08032516 | 0.922621903 | 1.264984549 | Malaysia | Male | 1975 to 1984 |
| 1985 | 1.075458546 | 0.964943363 | 1.19863106 | Malaysia | Male | 1980 to 1989 |
| 1990 | 1.058311952 | 0.968343043 | 1.156639887 | Malaysia | Male | 1985 to 1994 |
| 1995 | 1 | 1 | 1 | Malaysia | Male | 1990 to 1999 |
| 2000 | 1.041962215 | 0.956510083 | 1.135048419 | Malaysia | Male | 1995 to 2004 |
| 2005 | 1.189044113 | 1.085818481 | 1.302083108 | Malaysia | Male | 2000 to 2009 |
| 2010 | 1.371160081 | 1.230293558 | 1.528155581 | Malaysia | Male | 2005 to 2014 |
| 2015 | 1.535823274 | 1.319991853 | 1.786945217 | Malaysia | Male | 2010 to 2019 |
| 1980 | 1.130074728 | 1.02875258 | 1.241376124 | China | Male | 1975 to 1984 |
| 1985 | 1.0856489 | 1.016943903 | 1.158995625 | China | Male | 1980 to 1989 |
| 1990 | 1.045303975 | 0.991185138 | 1.102377707 | China | Male | 1985 to 1994 |
| 1995 | 1 | 1 | 1 | China | Male | 1990 to 1999 |
| 2000 | 0.967779785 | 0.911688697 | 1.027321843 | China | Male | 1995 to 2004 |
| 2005 | 1.034894121 | 0.972302687 | 1.101514844 | China | Male | 2000 to 2009 |
| 2010 | 1.213174328 | 1.133785013 | 1.298122601 | China | Male | 2005 to 2014 |
| 2015 | 1.379037578 | 1.26214261 | 1.506758924 | China | Male | 2010 to 2019 |
| 1980 | 0.853928968 | 0.74683602 | 0.976378568 | Viet Nam | Male | 1975 to 1984 |
| 1985 | 0.963766088 | 0.882098719 | 1.052994469 | Viet Nam | Male | 1980 to 1989 |
| 1990 | 1.036185412 | 0.967187573 | 1.110105462 | Viet Nam | Male | 1985 to 1994 |
| 1995 | 1 | 1 | 1 | Viet Nam | Male | 1990 to 1999 |
| 2000 | 0.932439559 | 0.867360384 | 1.002401709 | Viet Nam | Male | 1995 to 2004 |
| 2005 | 0.981103759 | 0.906980976 | 1.061284208 | Viet Nam | Male | 2000 to 2009 |
| 2010 | 1.110817734 | 1.011599131 | 1.219767792 | Viet Nam | Male | 2005 to 2014 |
| 2015 | 1.284821456 | 1.122161863 | 1.47105888 | Viet Nam | Male | 2010 to 2019 |
| 1980 | 0.979557367 | 0.917468032 | 1.045848576 | Philippines | Male | 1975 to 1984 |
| 1985 | 0.986263557 | 0.944061674 | 1.030351968 | Philippines | Male | 1980 to 1989 |
| 1990 | 0.990687841 | 0.95696756 | 1.025596311 | Philippines | Male | 1985 to 1994 |
| 1995 | 1 | 1 | 1 | Philippines | Male | 1990 to 1999 |
| 2000 | 0.970962146 | 0.938984086 | 1.004029252 | Philippines | Male | 1995 to 2004 |
| 2005 | 0.880996545 | 0.850308778 | 0.912791837 | Philippines | Male | 2000 to 2009 |
| 2010 | 0.790946654 | 0.759176193 | 0.824046663 | Philippines | Male | 2005 to 2014 |
| 2015 | 0.687658439 | 0.649865167 | 0.7276496 | Philippines | Male | 2010 to 2019 |
| 1980 | 0.989842982 | 0.921569588 | 1.063174329 | Global | Female | 1975 to 1984 |
| 1985 | 0.985406494 | 0.937288375 | 1.035994881 | Global | Female | 1980 to 1989 |
| 1990 | 0.987017828 | 0.946992075 | 1.028735318 | Global | Female | 1985 to 1994 |
| 1995 | 1 | 1 | 1 | Global | Female | 1990 to 1999 |
| 2000 | 0.990255297 | 0.950125202 | 1.032080353 | Global | Female | 1995 to 2004 |
| 2005 | 0.975711535 | 0.934784178 | 1.0184308 | Global | Female | 2000 to 2009 |
| 2010 | 0.957190713 | 0.910524223 | 1.00624897 | Global | Female | 2005 to 2014 |
| 2015 | 0.892433826 | 0.832707483 | 0.956444069 | Global | Female | 2010 to 2019 |
| 1980 | 1.083594631 | 1.00670385 | 1.166358235 | Western Pacific Region | Female | 1975 to 1984 |
| 1985 | 1.038134305 | 0.986057339 | 1.09296163 | Western Pacific Region | Female | 1980 to 1989 |
| 1990 | 0.994428083 | 0.953191839 | 1.037448258 | Western Pacific Region | Female | 1985 to 1994 |
| 1995 | 1 | 1 | 1 | Western Pacific Region | Female | 1990 to 1999 |
| 2000 | 1.007369465 | 0.96242437 | 1.05441349 | Western Pacific Region | Female | 1995 to 2004 |
| 2005 | 1.037751369 | 0.989574029 | 1.088274219 | Western Pacific Region | Female | 2000 to 2009 |
| 2010 | 1.126238116 | 1.068078389 | 1.187564797 | Western Pacific Region | Female | 2005 to 2014 |
| 2015 | 1.125387192 | 1.048500185 | 1.207912359 | Western Pacific Region | Female | 2010 to 2019 |
| 1980 | 1.067702962 | 0.96306904 | 1.183704976 | Japan | Female | 1975 to 1984 |
| 1985 | 1.017125369 | 0.94130801 | 1.099049415 | Japan | Female | 1980 to 1989 |
| 1990 | 1.003760966 | 0.938955258 | 1.073039496 | Japan | Female | 1985 to 1994 |
| 1995 | 1 | 1 | 1 | Japan | Female | 1990 to 1999 |
| 2000 | 1.000420807 | 0.934107208 | 1.071442104 | Japan | Female | 1995 to 2004 |
| 2005 | 0.976495972 | 0.907861409 | 1.050319327 | Japan | Female | 2000 to 2009 |
| 2010 | 0.969393873 | 0.889789477 | 1.05612002 | Japan | Female | 2005 to 2014 |
| 2015 | 0.959083336 | 0.844930343 | 1.08865879 | Japan | Female | 2010 to 2019 |
| 1980 | 0.899492957 | 0.821174495 | 0.985280941 | Singapore | Female | 1975 to 1984 |
| 1985 | 0.961790242 | 0.900766065 | 1.026948622 | Singapore | Female | 1980 to 1989 |
| 1990 | 0.9841324 | 0.933503853 | 1.037506784 | Singapore | Female | 1985 to 1994 |
| 1995 | 1 | 1 | 1 | Singapore | Female | 1990 to 1999 |
| 2000 | 0.991384194 | 0.940976304 | 1.044492423 | Singapore | Female | 1995 to 2004 |
| 2005 | 0.952941602 | 0.900412951 | 1.008534692 | Singapore | Female | 2000 to 2009 |
| 2010 | 0.951043779 | 0.88921904 | 1.017167006 | Singapore | Female | 2005 to 2014 |
| 2015 | 0.951337658 | 0.866869 | 1.044037034 | Singapore | Female | 2010 to 2019 |
| 1980 | 1.005985859 | 0.848066909 | 1.193310973 | Malaysia | Female | 1975 to 1984 |
| 1985 | 1.002959774 | 0.890469804 | 1.129660213 | Malaysia | Female | 1980 to 1989 |
| 1990 | 1.01211661 | 0.91648282 | 1.117729662 | Malaysia | Female | 1985 to 1994 |
| 1995 | 1 | 1 | 1 | Malaysia | Female | 1990 to 1999 |
| 2000 | 1.090241976 | 0.992232649 | 1.197932328 | Malaysia | Female | 1995 to 2004 |
| 2005 | 1.232532991 | 1.117246353 | 1.359715849 | Malaysia | Female | 2000 to 2009 |
| 2010 | 1.422417901 | 1.266595601 | 1.597410162 | Malaysia | Female | 2005 to 2014 |
| 2015 | 1.588167049 | 1.354956247 | 1.861517359 | Malaysia | Female | 2010 to 2019 |
| 1980 | 1.128675899 | 0.988352505 | 1.288921997 | China | Female | 1975 to 1984 |
| 1985 | 1.107835758 | 1.009216626 | 1.216091805 | China | Female | 1980 to 1989 |
| 1990 | 1.070980825 | 0.992570423 | 1.15558544 | China | Female | 1985 to 1994 |
| 1995 | 1 | 1 | 1 | China | Female | 1990 to 1999 |
| 2000 | 0.932966117 | 0.853998703 | 1.019235477 | China | Female | 1995 to 2004 |
| 2005 | 1.004632743 | 0.917187653 | 1.100414888 | China | Female | 2000 to 2009 |
| 2010 | 1.205715818 | 1.094629361 | 1.328075682 | China | Female | 2005 to 2014 |
| 2015 | 1.327174911 | 1.173322944 | 1.501200716 | China | Female | 2010 to 2019 |
| 1980 | 0.906941833 | 0.791861037 | 1.038747267 | Viet Nam | Female | 1975 to 1984 |
| 1985 | 0.99528553 | 0.910309584 | 1.088193844 | Viet Nam | Female | 1980 to 1989 |
| 1990 | 1.045801345 | 0.975442575 | 1.121235101 | Viet Nam | Female | 1985 to 1994 |
| 1995 | 1 | 1 | 1 | Viet Nam | Female | 1990 to 1999 |
| 2000 | 0.929799271 | 0.864112189 | 1.000479677 | Viet Nam | Female | 1995 to 2004 |
| 2005 | 0.967772357 | 0.894421059 | 1.047139181 | Viet Nam | Female | 2000 to 2009 |
| 2010 | 1.070195196 | 0.975491668 | 1.174092815 | Viet Nam | Female | 2005 to 2014 |
| 2015 | 1.177417623 | 1.03228405 | 1.342956193 | Viet Nam | Female | 2010 to 2019 |
| 1980 | 1.202248024 | 1.117749352 | 1.293134555 | Philippines | Female | 1975 to 1984 |
| 1985 | 1.116553165 | 1.063458111 | 1.172299086 | Philippines | Female | 1980 to 1989 |
| 1990 | 1.019224283 | 0.980422913 | 1.059561262 | Philippines | Female | 1985 to 1994 |
| 1995 | 1 | 1 | 1 | Philippines | Female | 1990 to 1999 |
| 2000 | 0.995941856 | 0.959155 | 1.034139614 | Philippines | Female | 1995 to 2004 |
| 2005 | 0.925535299 | 0.88965873 | 0.962858634 | Philippines | Female | 2000 to 2009 |
| 2010 | 0.832659599 | 0.79597407 | 0.871035922 | Philippines | Female | 2005 to 2014 |
| 2015 | 0.710990915 | 0.668744243 | 0.755906442 | Philippines | Female | 2010 to 2019 |
| 1980 | 0.97299706 | 0.904282781 | 1.046932772 | Global | Both | 1975 to 1984 |
| 1985 | 0.972414086 | 0.924001133 | 1.023363632 | Global | Both | 1980 to 1989 |
| 1990 | 0.983945987 | 0.943425651 | 1.02620668 | Global | Both | 1985 to 1994 |
| 1995 | 1 | 1 | 1 | Global | Both | 1990 to 1999 |
| 2000 | 0.994571 | 0.953817001 | 1.037066307 | Global | Both | 1995 to 2004 |
| 2005 | 0.988154188 | 0.946067044 | 1.03211364 | Global | Both | 2000 to 2009 |
| 2010 | 0.97286258 | 0.924691347 | 1.023543264 | Global | Both | 2005 to 2014 |
| 2015 | 0.923579996 | 0.860509079 | 0.991273689 | Global | Both | 2010 to 2019 |
| 1980 | 1.088649278 | 1.018244586 | 1.163921976 | Western Pacific Region | Both | 1975 to 1984 |
| 1985 | 1.040953701 | 0.993687185 | 1.090468534 | Western Pacific Region | Both | 1980 to 1989 |
| 1990 | 1.000454062 | 0.963180489 | 1.039170063 | Western Pacific Region | Both | 1985 to 1994 |
| 1995 | 1 | 1 | 1 | Western Pacific Region | Both | 1990 to 1999 |
| 2000 | 0.994848955 | 0.955076745 | 1.036277397 | Western Pacific Region | Both | 1995 to 2004 |
| 2005 | 1.020814048 | 0.978020647 | 1.06547988 | Western Pacific Region | Both | 2000 to 2009 |
| 2010 | 1.108879932 | 1.056981222 | 1.163326914 | Western Pacific Region | Both | 2005 to 2014 |
| 2015 | 1.128227133 | 1.057913439 | 1.203214191 | Western Pacific Region | Both | 2010 to 2019 |
| 1980 | 1.521984608 | 1.471992143 | 1.57367494 | Japan | Both | 1975 to 1984 |
| 1985 | 1.290527837 | 1.259398174 | 1.32242696 | Japan | Both | 1980 to 1989 |
| 1990 | 1.125408493 | 1.102569771 | 1.148720299 | Japan | Both | 1985 to 1994 |
| 1995 | 1 | 1 | 1 | Japan | Both | 1990 to 1999 |
| 2000 | 0.931981911 | 0.911829922 | 0.952579272 | Japan | Both | 1995 to 2004 |
| 2005 | 0.874041403 | 0.85359854 | 0.894973854 | Japan | Both | 2000 to 2009 |
| 2010 | 0.799698925 | 0.777644492 | 0.822378834 | Japan | Both | 2005 to 2014 |
| 2015 | 0.720128469 | 0.690780444 | 0.750723353 | Japan | Both | 2010 to 2019 |
| 1980 | 0.983214255 | 0.927500429 | 1.042274742 | Singapore | Both | 1975 to 1984 |
| 1985 | 1.003095359 | 0.961542621 | 1.046443784 | Singapore | Both | 1980 to 1989 |
| 1990 | 1.011158758 | 0.978049581 | 1.045388755 | Singapore | Both | 1985 to 1994 |
| 1995 | 1 | 1 | 1 | Singapore | Both | 1990 to 1999 |
| 2000 | 0.977821799 | 0.945688705 | 1.011046728 | Singapore | Both | 1995 to 2004 |
| 2005 | 0.961187253 | 0.927497298 | 0.996100945 | Singapore | Both | 2000 to 2009 |
| 2010 | 0.93283355 | 0.894360699 | 0.972961393 | Singapore | Both | 2005 to 2014 |
| 2015 | 0.938793971 | 0.887062096 | 0.993542757 | Singapore | Both | 2010 to 2019 |
| 1980 | 1.043857226 | 0.887757302 | 1.227405178 | Malaysia | Both | 1975 to 1984 |
| 1985 | 1.040725368 | 0.930449369 | 1.164071177 | Malaysia | Both | 1980 to 1989 |
| 1990 | 1.03712077 | 0.945461374 | 1.137666245 | Malaysia | Both | 1985 to 1994 |
| 1995 | 1 | 1 | 1 | Malaysia | Both | 1990 to 1999 |
| 2000 | 1.063068509 | 0.97300478 | 1.161468761 | Malaysia | Both | 1995 to 2004 |
| 2005 | 1.208457158 | 1.100953122 | 1.32645857 | Malaysia | Both | 2000 to 2009 |
| 2010 | 1.395480779 | 1.249288087 | 1.558781055 | Malaysia | Both | 2005 to 2014 |
| 2015 | 1.56281027 | 1.340944827 | 1.821384363 | Malaysia | Both | 2010 to 2019 |
| 1980 | 1.122820968 | 1.008337586 | 1.250302421 | China | Both | 1975 to 1984 |
| 1985 | 1.08837445 | 1.009607244 | 1.173286889 | China | Both | 1980 to 1989 |
| 1990 | 1.052129802 | 0.989699938 | 1.118497716 | China | Both | 1985 to 1994 |
| 1995 | 1 | 1 | 1 | China | Both | 1990 to 1999 |
| 2000 | 0.955880898 | 0.891593068 | 1.024804168 | China | Both | 1995 to 2004 |
| 2005 | 1.024879195 | 0.953352685 | 1.101772073 | China | Both | 2000 to 2009 |
| 2010 | 1.21066223 | 1.12001139 | 1.308650117 | China | Both | 2005 to 2014 |
| 2015 | 1.358266464 | 1.227997608 | 1.502354544 | China | Both | 2010 to 2019 |
| 1980 | 0.871779495 | 0.761913792 | 0.997487505 | Viet Nam | Both | 1975 to 1984 |
| 1985 | 0.974547963 | 0.89171113 | 1.065080047 | Viet Nam | Both | 1980 to 1989 |
| 1990 | 1.039976854 | 0.970422049 | 1.114516986 | Viet Nam | Both | 1985 to 1994 |
| 1995 | 1 | 1 | 1 | Viet Nam | Both | 1990 to 1999 |
| 2000 | 0.930976462 | 0.865663541 | 1.001217138 | Viet Nam | Both | 1995 to 2004 |
| 2005 | 0.975804012 | 0.901995435 | 1.055652205 | Viet Nam | Both | 2000 to 2009 |
| 2010 | 1.094304665 | 0.996934247 | 1.201185237 | Viet Nam | Both | 2005 to 2014 |
| 2015 | 1.238492793 | 1.083423543 | 1.415756938 | Viet Nam | Both | 2010 to 2019 |
| 1980 | 1.076465665 | 1.030067838 | 1.124953411 | Philippines | Both | 1975 to 1984 |
| 1985 | 1.044354165 | 1.014055916 | 1.075557674 | Philippines | Both | 1980 to 1989 |
| 1990 | 1.003062682 | 0.979879478 | 1.026794384 | Philippines | Both | 1985 to 1994 |
| 1995 | 1 | 1 | 1 | Philippines | Both | 1990 to 1999 |
| 2000 | 0.98303083 | 0.961028927 | 1.005536447 | Philippines | Both | 1995 to 2004 |
| 2005 | 0.901947726 | 0.880674303 | 0.923735026 | Philippines | Both | 2000 to 2009 |
| 2010 | 0.810570348 | 0.788649584 | 0.833100407 | Philippines | Both | 2005 to 2014 |
| 2015 | 0.697932665 | 0.672204724 | 0.724645317 | Philippines | Both | 2010 to 2019 |
